# Non-ribosomal phylogenetic exploration of *Mollicute* species: New insights into haemoplasma taxonomy

**DOI:** 10.1016/j.meegid.2014.02.001

**Published:** 2014-04

**Authors:** C.A.E. Hicks, E.N. Barker, C. Brady, C.R. Stokes, C.R. Helps, S. Tasker

**Affiliations:** a School of Veterinary Sciences, University of Bristol, Langford BS40 5DU, United Kingdom; b Department of Applied Sciences, University of the West of England, Frenchay Campus, Coldharbour Lane, Bristol BS16 1QY, United Kingdom

**Keywords:** Mycoplasma, Phylogeny, *gapA*, *dnaK*, Hemotropic Mycoplasmas

## Abstract

Nine species of uncultivable haemoplasmas and several *Mycoplasma* species were examined by partial sequencing of two protein-encoding housekeeping genes. Partial glyceraldehyde-3-phosphate dehydrogenase (*gapA*) and heat shock protein 70 (*dnaK*) gene sequences were determined for these *Mollicute* species; in total nine *gapA* sequences and ten *dnaK* sequences were obtained. Phylogenetic analyses of these sequences, along with those of a broad selection of *Mollicute* species downloaded from GenBank, for the individual genes, and for the *gapA* and *dnaK* concatenated data set, revealed a clear separation of the haemoplasmas from other species within the *Mycoplasma* genus; indeed the haemoplasmas resided within a single clade which was phylogenetically detached from the pneumoniae group of Mycoplasmas. This is the first report to examine the use of *gapA* and *dnaK*, as well as a concatenated data set, for phylogenetic analysis of the haemoplasmas and other *Mollicute* species. These results demonstrate a distinct phylogenetic separation between the haemoplasmas and Mycoplasmas that corresponds with the biological differences observed in these species, indicating that further evaluation of the haemoplasmas’ relationship with the *Mycoplasma* genus is required to determine whether reclassification of the haemoplasmas is necessary.

## Highlights

• The first *gapA* and *dnaK* phylogenetic analysis of *Mollicute* species.
• Analysis includes a wide range of haemoplasmas species.
• Analysis supports that haemoplasmas reside within a single clade.
• Evidence of phylogenetic distance between the haemoplasmas and *Mycoplasma* species.

## 1 Introduction

The taxonomic position of the *Eperythrozoon* and *Haemobartonella* species has long been a subject of controversy. Originally classified within the order *Rickettsiales*, they were reclassified as members of the class *Mollicutes*, order *Mycoplasmatales* and family *Mycoplasmataceae*, genus *Mycoplasma* (Brown et al., 2010b) or family *Incertae Sedis*, genus *Eperythrozoon* or *Haemobartonella* (Brown et al., 2010a), on the basis of 16S rRNA gene sequence analysis, and given the trivial name haemoplasma (Messick et al., 2002; Neimark et al., 2001, 2002; Rikihisa et al., 1997). Phylogenetic characterisation using the RNaseP RNA (*rnpB*) gene has supported the 16S rDNA-based phylogeny and shown that the haemoplasmas reside in a single clade, within the genus *Mycoplasma*, most closely related to the pneumoniae group of Mycoplasmas, with *Mycoplasma fastidiosum* and *Mycoplasma cavipharyngis* being their closest relatives (Johansson et al., 1999; Neimark et al., 2001; Peters et al., 2008; Rikihisa et al., 1997; Tasker et al., 2003). Haemoplasmas are, as yet, uncultivatable bacteria, limiting their phenotypic characterisation. They adhere to red blood cells causing varying degrees of anaemia, and can infect a large range of mammalian species including, but not limited to, cats (Foley and Pedersen, 2001; Tasker et al., 2009; Willi et al., 2005), dogs, alpacas, opossums (Messick et al., 2002), sheep, goats (Neimark et al., 2004), and humans (Steer et al., 2011).

Dispute over the nomenclature and classification of the haemoplasmas as members of the genus *Mycoplasma* has left many of them within the order *Mycoplasmatales*, family *Incertae sedis* under the genus *Eperythrozoon* or *Haemobartonella*; *Incertae sedis* being a taxonomic description given to species whose position and relationship with other species is undefined (Brown et al., 2010a; Neimark et al., 2005; Uilenberg et al., 2006). Indeed, an insufficient level of similarity to justify the classification of the haemoplasmas within the genus *Mycoplasma* was reported by Uilenberg et al. (2004)). Uilenberg et al. (2004) highlighted that only 77.3% 16S rRNA gene identity existed between *Mycoplasma wenyonii* (a haemoplasma species) and *M. fastidiosum* (a member of the genus *Mycoplasma*), and that significant differences in biological characteristics (e.g. biological niche, transmission methods, ability to culture *in vitro*) between the haemoplasmas and members of the genus *Mycoplasma* also existed.

Despite wide use of 16S rRNA gene and *rnpB* sequences to describe phylogenetic relationships between species of bacteria, both genes lack resolving power at the species level as they are highly conserved (Birkenheuer et al., 2002; Mignard and Flandrois, 2006; Stackebrandt and Goebel, 1994; Tasker et al., 2003). The *rnpB* sequence used in a previous haemoplasma phylogeny study showed little variation and was too short to give high bootstrap values (Peters et al., 2008). The use of multilocus sequence analysis (MLSA) of protein encoding genes has been proven to be useful in the determination of the taxonomic position of many bacteria. This approach has been previously used to analyse members of the *Mycoplasma* genus, using genes such as *tufA*, *fusA*, *gyrB*, *lepA*, *rpoB*, *efp*, *gmk* and *adk* (Kamla et al., 1996; Manso-Silván et al., 2012; Manso-Silván et al., 2007; Thompson et al., 2011). It was reported that *tufA* was more able to demonstrate the phenotypic features of the bacteria than the 16S rRNA gene, and MLSA proved useful for discrimination at sub-species levels. *gapA* and *dnaK* are two protein-encoding housekeeping genes that have been previously used in phylogenetic analysis of other bacteria due to their identification as good taxonomic markers (Falah and Grupta, 1997; Fraga et al., 2010; Martens et al., 2008; Wertz et al., 2003). Both *gapA* and *dnaK* should provide more resolving power than the 16S rRNA gene and *rnpB* as they are highly conserved across species but offer higher variation within the sequences than those of rRNA genes, and are well over twice the length of the *rnpB* gene; *gapA* and *dnaK* are approximately 1 Kbp and 1.8 Kbp respectively, in comparison to approximately 0.4 Kbp for *rnpB.*

The continued incorporation of the haemoplasmas within the order *Mycoplasmatales* family, *Incertae sedis* highlights the need to further explore the taxonomic position of these bacteria. This is the first report to examine the use of *gapA* and *dnaK* for phylogenetic analysis of a wide range of haemoplasmas and other *Mollicute* species, and furthermore the first to describe a concatenated data set for these genes in these species.

## 2 Materials and methods

### 2.1 Source of species

The samples used in the current study were DNA derived from species obtained for a previous study (Peters et al., 2008): *Mycoplasma coccoides*, *Mycoplasma haemomuris*, ‘*Candidatus* Mycoplasma haemolamae’, ‘*Candidatus* Mycoplasma kahaneii’, ‘*Candidatus* Mycoplasma haemocervae’, ‘*Candidatus* Mycoplasma haematoparvum’, ‘*Candidatus* Mycoplasma haemohominis’, ‘*Candidatus* Mycoplasma erythrocervae’, *Mycoplasma ovis*, *Mycoplasma felis,* and *M. fastidiosum*. Additionally, EDTA blood samples of *M. wenyonii, Mycoplasma haemomuris*, ‘*Candidatus* Mycoplasma erythrocervae’, ‘*Candidatus* Mycoplasma haemocervae’ and ‘*Candidatus* Mycoplasma haemohominis’ were obtained from clinical and experimentally infected cases, and a vial of *M. cavipharyngis* colonies on agar was kindly provided by Mycoplasma Experience (Reigate, UK).

### 2.2 DNA extraction

Genomic DNA was extracted from EDTA blood using the Nucleospin® Blood Kit (Macherey-Nagel) following the manufacturer’s protocol, eluting into 100 μl of buffer BE. For *M. cavipharyngis,* the agar sample was spun at 600*g* for 30 s and 100 μl of supernatant was then subjected to DNA extraction using the Nucleospin® Blood Kit as for the blood samples. DNA was stored at −20 °C until further use.

### 2.3 Primer design

Primers (Table 1) were designed for the amplification and sequencing of partial *gapA* and *dnaK* gene sequences using Primer3 v. 0.4.0 (Rozen and Skaletsky, 2000) and alignments of selected available haemoplasma and *Mycoplasma* sequences downloaded from GenBank (National Centre for Biotechnology Information, USA).

### 2.4 Polymerase chain reaction

Polymererase chain reaction (PCR) to amplify both *gapA* and *dnaK* was performed using DNA for all species and a combination of primers from Table 1. Each PCR reaction consisted of 12.5 μl of 2 X HotStar*Taq* Mastermix (Qiagen), MgCl_2_ to a final concentration of 4.5 mM, primers (200 nM for *dnaK* primers F34, R1139, R1367, and R1802; 400 nM for *dnaK* primers F61, F146, F603, R874, and R1052; 200 nM for *gapA* primers F22, F369 and R975; 400 nM for *gapA* primers F27, F71, R667, R683, R729, and R968), 1 μl of template DNA and water to a final volume of 25 μl. A positive control (*M. haemofelis*/‘*Ca.* M. haemominutum’) and a negative control (water) were run alongside the samples in all PCR runs. A MJ Research PTC-200 Peltier thermal cycler (Bio-Rad) was used for PCR, set to incubate at 95 °C for 15 min, then 45 cycles of 95 °C for 10 s, 50 °C for 15 s and 72 °C for 90 s, followed by 72 °C for 15 min. Products were separated on a 1% agarose gel, and products of the appropriate size were purified using NucleoSpin® Extract II Kit (Machery-Nagel). The amount of DNA present in each sample was quantified using the Quant-iT™ dsDNA Broad-Range or High-Sensitivity Assay Kits (Invitrogen) according to the manufacturer’s instructions. Re-amplification was carried out for reactions producing little product, using the procedure described above with 1 μl of PCR product as template. Samples were submitted to the DNA Sequencing & Services (MRCPPU, College of Life Sciences, University of Dundee, www.dnaseq.co.uk) for sequencing using an Applied Biosystems model 3730 automated capillary DNA sequencer after being diluted to a specified concentration dependant on amplicon size. Primers were added at a concentration of 3.2 μM to the samples to be sequenced.

### 2.5 Sequence analysis and phylogenetic analysis

Forward and reverse sequences for each sample were assembled using ClustalW in MacVector (MacVector and Assembler 11.1.2) and primer sequence sites were removed. Each sample was sequenced twice in both forward and reverse directions, and all sequences derived from each sample aligned to resolve any discrepancies. The final sequences for all samples were aligned using MAFFT version 7 (Katoh and Standley, 2013), along with selected haemoplasma and other *Mollicute dnaK* and *gapA* sequences available online in GenBank. The section of each gene for which sequence data were available for all samples was then subjected to phylogenetic analysis.

To establish that the two data sets, *gapA* and *dnaK*, could be combined a partition homogeneity test was run using Paup 4.0 (Swofford, 2003). Modeltest 3.7 (Posada and Crandall, 1998) was applied to the data to determine the model best suited for both the single gene data and the concatenated data set, for both aligned gene sequences and the concatenated aligned sequences the best fit evolutionary model determined by Modeltest 3.7 was the generalised time reversible model. The result of the modeltest was then used in Phyml 3.0 (Guindon et al., 2010) to generate maximum likelihood trees viewed using Treeview (Page, 1996). In addition, neighbour-joining trees were constructed using the Kimura-2 parameter model in Mega5 (Tamura et al., 2011).

Bootstrap analysis of the trees was preformed to 1000 replicates and *Clostridium perfringens* (GenBank: BA000016) was chosen as the out-group.

## 3 Results and discussion

This study represents the first use of the housekeeping genes *gapA* and *dnaK*, as well as a concatenated data analysis, for a wide range of haemoplasma and other *Mollicute* species. Partial *gapA* and *dnaK* gene sequences were obtained for most species, as shown in Table 2. Only partial sequences could be obtained for the two genes; sequence lengths of 466 bp and 509 bp for *gapA* and *dnaK*, respectively, were analysed phylogenetically to produce maximum likelihood individual and concatenated trees. Nucleotide sequence data generated from this study are available from the GenBank database (Genbank: KF151041-151059).

**Fig. 1:**
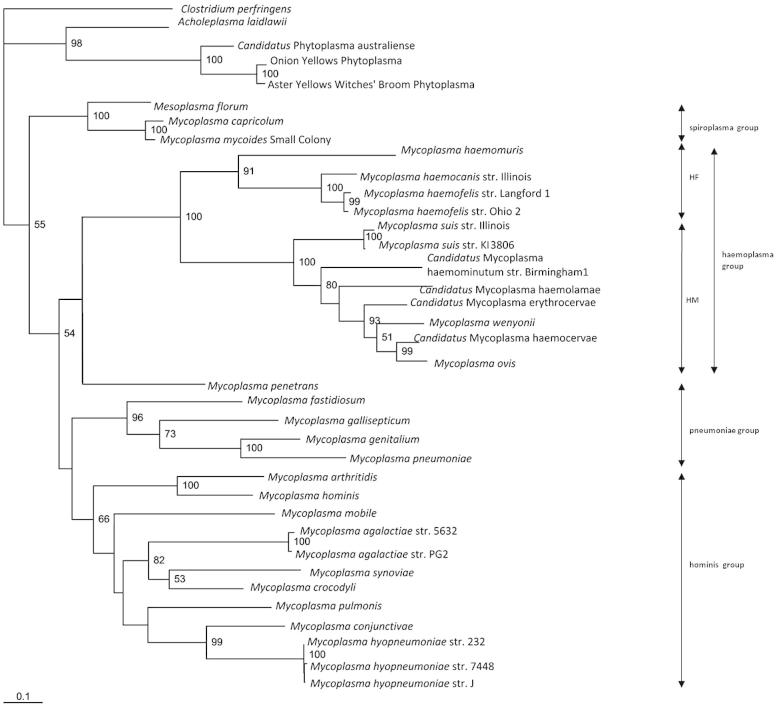
Concatenated maximum likelihood tree for *dnaK* and *gapA*. *DnaK* and *gapA* sequences from the *Mollicute* species were added together to create a concatenated data set, and a maximum likelihood tree was constructed from this. Mycoplasma groupings (hominis group, spiroplasma group, and pneumoniae group) are given as previously described (Peters et al., 2008; Weisburg et al., 1989) and the haemoplasmas are also specified; HF indicates the haemofelis subgroup and HM indicates the haemominutum subgroup. The data set was resampled 1000 times and the resulting bootstrap values are given as percentages at the nodes (values less than 50% are not shown).

**Fig. 2:**
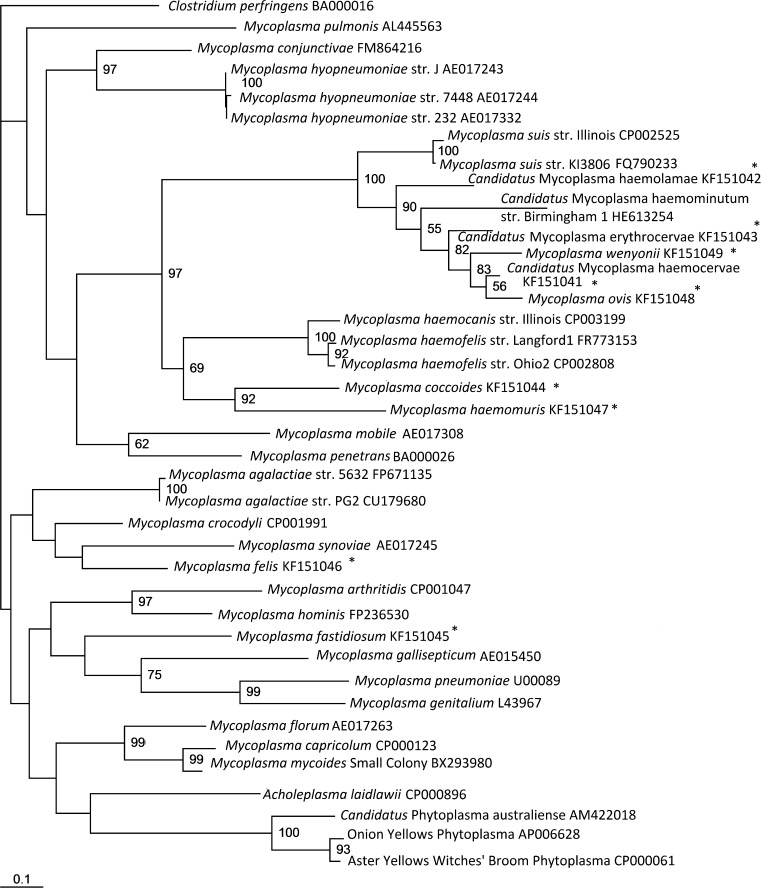
Maximum likelihood tree for *gapA* sequences. A *gapA* sequence length of 466 bp was analysed for a number of haemoplasma and other *Mollicute* species, and a maximum likelihood tree was constructed. ^*^Indicates sequences derived in the current study. Accession numbers are given. The data set was resampled 1000 times and the resulting bootstrap values are given as percentages at the nodes (values less than 50% are not shown).

**Fig. 3:**
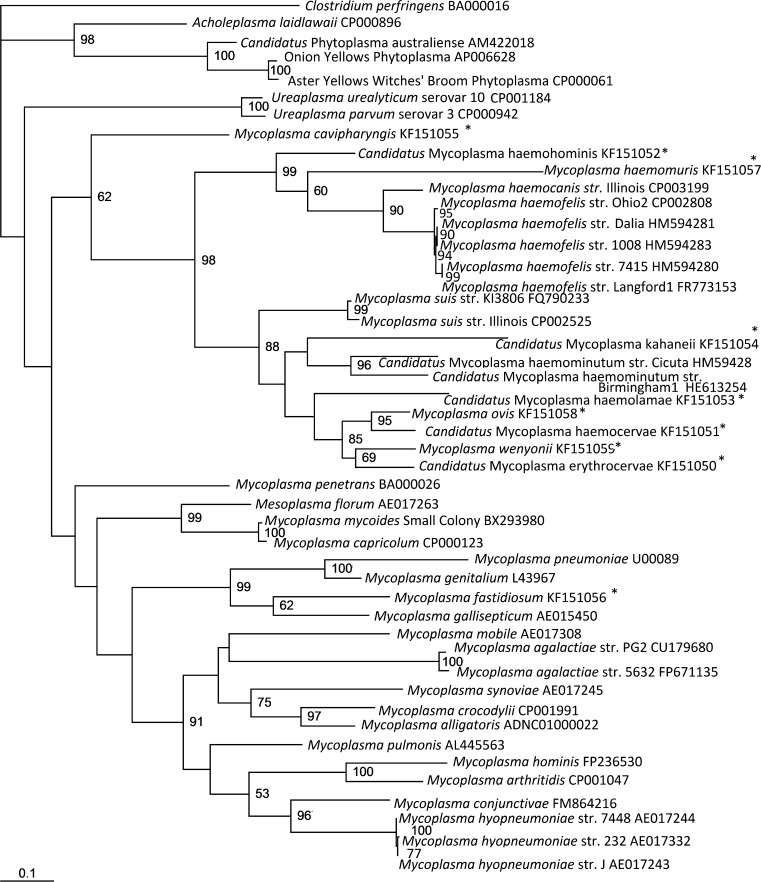
Maximum likelihood tree of *dnaK* sequences. A *dnaK* sequence length of 509 bp was analysed for a number of haemoplasma and other *Mollicute* species, and a maximum likelihood tree was constructed. ^*^Indicates sequences derived as part of the current study. Accession numbers are given. The data set was resampled 1000 times and the resulting bootstrap values are given as percentages at the nodes (values less than 50% are not shown).

Considering firstly the non-haemoplasma *Mycoplasma* species, the concatenated tree, as shown in Fig. 1, separates the non-haemoplasma *Mycoplasma* genus species into three separate groups: the hominis group, the pneumoniae group and the spiroplasma group, which is consistent with the 16S rRNA-based phylogenetic analysis of *Mycoplasma* species (Weisburg et al., 1989). Additionally, species of the order *Acholeplasmatales* (including the *Acholeoplasma* and *Phytoplasma* species) were separated from those of the order *Mycoplasmatales*. It is apparent in all the trees produced in this study (Figs. 1–3) that the type species for the *Mycoplasma* genus, *Mycoplasma mycoides*, as well as *Mycoplasma capricolum*, cluster closely with *Mesoplasma florum* of the order *Entoplasmatales*, family *Entoplasmataceae*, genus *Mesoplasma*. This further supports previous work suggesting that *M. mycoides* and *M. capricolum* do not belong in the order *Mycoplasmatales* and family *Mycoplasmataceae*, let alone the same genus as the other *Mycoplasma* species (Weisburg et al., 1989). However, despite the evidence these species remain within the genus *Mycoplasma* due to the confusion that reclassification of the type species outside of this genus would cause within the scientific community; re-naming of all other *Mycoplasma* species would be needed if this occurred, following the identification of a new type species for the *Mycoplasma* genus (Brown et al., 2010b; Gasparich et al., 2004; Tully et al., 1993).

It is evident from the concatenated tree (Fig. 1) that there is a distinct separation of the haemoplasmas from the other *Mycoplasma* species, supported by a bootstrap value of 100%, whilst the pneumoniae and hominis groups of the genus *Mycoplasma* reside in a separate clade. This observation has not been described before for haemoplasma phylogeny using 16S rDNA- and *rnpB*-based phylogeny (Johansson et al., 1999; Neimark and Kocan, 1997; Peters et al., 2008; Rikihisa et al., 1997). The construction of a neighbour-joining tree (data not shown) confirmed the separate clustering of the haemoplasma species from the other genus *Mycoplasma* species. The high level of support for this cluster is confirmed in both the individual gene trees (Figs. 2 and 3). This division of the haemoplasma species is most likely accounted for by the distinct biological differences between the haemoplasmas and other members of the *Mycoplasma* genus. The natural habitat of the Mycoplasmas is usually the mucosal surfaces of the respiratory and urogenital tracts of vertebrate species, as well as the eyes and joints (Razin et al., 1998), conversely the haemoplasmas reside attached to red blood cells. Other biological differences between the haemoplasmas and members of the genus *Mycoplasma* include the haemoplasmas’ suspected arthropod transmission, with haemoplasma DNA being found in both fleas and ticks (Woods et al., 2005; Woods et al., 2006), and the haemoplasmas’ lack of ability to grow in an *in vitro* system. Despite multiple attempts, including the use of *Mycoplasma*-specific media, the haemoplasmas remain one of the few Mycoplasmas currently uncultivated *in vitro*, highlighting a specific growth requirement of the haemoplasmas not seen in the other species of the genus *Mycoplasma*. Additionally, as previously described, there is a lack of identity between the haemoplasmas and species of the genus *Mycoplasma* (Uilenberg et al., 2004, 2006).

Our study shows that there is also considerable distance between the haemoplasmas and their closest relatives, *Mycoplasma penetrans, M. fastidiosum, M. cavipharyngis* and the other members of the pneumoniae group of Mycoplasmas. The concatenated tree does show the haemoplasmas sharing a node with *M. penetrans*, but there is no support given to this relationship due to its low bootstrap value (41.5%) (Fig. 1). This is in contrast to the closer relationship of the haemoplasmas to the pneumoniae group reported previously (Johansson et al., 1999; Peters et al., 2008; Tasker et al., 2003). Although the concatenated tree still shows the pneumoniae group to be the haemoplasmas’ closest relatives, the phylogenetic separation between these species is great enough to suggest that the haemoplasmas should comprise a separate distinct genus. Messick et al. (2002) have also reported that the haemoplasmas are missing some 16S rRNA residues and folding patterns which define the pneumoniae group, suggesting that the haemoplasmas are only peripherally linked to the pneumoniae group.

The derived concatenated maximum likelihood tree (Fig. 1) confirmed that the haemoplasma species reside within a single clade consisting of two subgroups: the so-called haemofelis cluster (consisting of *M. haemofelis, M*. *haemocanis,* and *M. haemomuris*) and the so-called haemominutum cluster (consisting of *‘Ca.* M. haemominutum’, *Mycoplasma suis, ‘Ca.* M. haemolamae’, ‘*Ca.* M. erythrocervae’, ‘*Ca.* M. haemocervae’, *M. wenyonii*, and *M. ovis*), as has been described by Peters et al for both 16S rDNA- and *rnpB*-based phylogeny (Peters et al., 2008). Both individual trees (Figs. 2 and 3) also support that the haemoplasma clade consists of two subgroups (Peters et al., 2008; Tasker et al., 2003). Like *rnpB*-based phylogeny, both *gapA* and *dnaK* were able to discriminate between the closely related *M. haemofelis* and *M. haemocanis* (Birkenheuer et al., 2002), which 16S rRNA gene-based phylogeny has failed to do (Birkenheuer et al., 2002).

Here we report the use of *gapA* and *dnaK* sequences for the analysis of the phylogenetic relationships of the haemoplasmas within the *Mollicutes*. Our work has shown that the resulting phylogeny using these non-ribosomal genes clearly differentiates the haemoplasmas and other species of the *Mycoplasma* genus into separate clades, divides the haemoplasmas into the previously reported haemofelis and haemominutum subgroups, and distinguishes between the haemoplasma species, especially the closely related species *M. haemocanis* and *M. haemofelis*. The separation of the haemoplasmas from those of the *Mycoplasma* genus strongly indicates that these results correspond with the differences in biological characteristics of these bacteria, suggesting that the haemoplasmas may not be as closely related to the *Mycoplasma* species as has been previously reported. These results suggest the possibility that the haemoplasmas could reside within their own genus, but further analysis, using more genes, would be required to determine whether this is true. We suggest that the taxonomic position of these bacteria may be better evaluated by MLSA, and that further evaluation may provide support for a new genus for the haemoplasmas.

## Figures and Tables

**Table 1:** Primers for the amplification and sequencing of *gapA* and *dnaK* partial sequences.

Primer	Primer sequence 5′-3′
*dnaK*	
F34	GACCTAGGTACAACTAACTCYTGTG
F61	TWGGTGGTGATGATTGRGA
F146	GGDGGAGGWACWTTTGAYG
F350	GTTATTACTGTTCCAGCATACTTTAA
F603	DGGRGGWACWTTTGAYGTYT
R874	CKCCCTGWACTACRTGAATRTCT
R1052	ATTCKWGTWGAWCCHCCDAC
R1139	CCACCTAGTGTTTCAATACTTAGAGTT
R1367	CCGTTAGCGTCAATAGAGAAGG
R1802	TTAGTTTTATCTACCTCAGTCTTATCCT

*gapA*	
F22	GGATTCGGAAGAATCGGAAG
F27	TGGATTYGGAAGAATMGGWAG
F71	AATGGHTTYGGWMGDATYGG
F369	AGTTATCTCCGCTCCAGCAAA
R667	GGWGCATCHTGWADTYTTTG
R683	TWCCWATWGCNGCAGAWGCWCCKGT
R729	ACTCTRTGHGCAATHCCATC
R968	TGRYTNACATAAGAAGAYTCRTTATCRTA
R975	AACAAGCTGATTCACATAAGAAGA

**Table 2:** *GapA* and *dnaK* accession numbers for all sequences obtained in this study.

Sample	GapA	DnaK
*‘Ca.* M. haemolamae’	KF151042	KF151053
‘*Ca.* M. haemohominis’	No amplification	KF151052
*‘Ca*. M. Kahaneii’	No amplification	KF151054
*‘Ca*. M. erythrocervae’	KF151043	KF151050
*‘Ca*. M. haemocervae’	KF151041	KF151051
*M. coccoides*	KF151044	No amplification
*M. ovis*	KF151048	KF151058
*M. wenyonii*	KF151049	KF151059
*M. haemomuris*	KF151047	KF151057
*M. cavipharyngis*	No amplification	KF151055
*M. fastidiosum*	KF151045	KF151056
*M. felis*	KF151046	No amplification

For some samples only *gapA* or *dnaK* sequences could be amplified; these samples were thus not included in the concatenated data set. Attempts to amplify and sequence full length *gapA* and *dnaK* sequences from all species were unsuccessful; partial gene sequences were generated, and the length for which there was overlap in all species was subjected to phylogenetic analysis, corresponding to 466 bp for *gapA* and 509 bp for *dnaK*.

## Appendix A Supplementary material

Supplementary figure A Fig. A. *gapA* sequence alignment.

Supplementary figure B Fig. B. *dnaK* sequence alignment.

Supplementary figure C Fig. C. Concatenated sequence alignment.
