# Supplementary material for: Non-ribosomal phylogenetic exploration of Mollicute species: New insights into haemoplasma taxonomy
Source: Infect Genet Evol. 2014 Apr;23(100):99–105. doi: 10.1016/j.meegid.2014.02.001 (PMC3988868; doi:10.1016/j.meegid.2014.02.001)
Supplement: Supplementary figure A — Fig. A. gapA sequence alignment. [file mmc1.pdf]

Supplementary Fig. A: GapA sequence alignment

466bp of *gapA* sequence for all Mollicute species in this study, aligned using MAFFT online multiple sequence alignment programme. Sequences are given in fasta format.

>*Mycoplasma pneumoniae* U00089

TAACGACCTCACCCATCCCGATACTTTAGCACACTTACTAAAGTACGACTCCGCTCACGGTGAGTT---
TAAGAAAAAGGTAGTA------------GCTAAGGACAACACCTTAATGATTGAC------------------
AAAAAGAAGGTGTTAGTGTTTAGTGAAAAAGATCCTGCTAACCTGCCATGAGCAGAACACAATATTGATATTG
TGGTTGAATCCACTGGTCGCTTTGTTAGTGAAGAAGGTGCTAGTTTGCACTTGCAAGCTGGTGCTAAACGCGT
GATTATTTCCGCTCCAGCTAAACAAAAAACAATTAAAACCGTGGTTTACAACGTGAACCACAAGATTATTAACG
CTGAGGACAAAATTATCTCAGCTGCTAGCTGTACCACCAATTGCTTAGCACCAATGGTACACGTCTTGGAAAA
GAACTTCGGCATCTTACACGGTACCATG

> *Mycoplasma genitalium* L43967

TAATGATTTGACCCAACCTGAAGTTTTAGCGCACCTGTTGAAATATGATTCAGCTCATGGTGAATT---
GAAAAGAAAGATTACT------------GTTAAACAAAACATCTTGCAAATTGAT------------------
AGAAAAAAGGTTTATGTTTTTAGTGAAAAAGATCCCCAAAATTTACCTTGGGATGAACATGATATTGATGTAG
TAATTGAATCAACTGGTAGGTTTGTAAGTGAAGAGGGTGCTTCTCTCCATTTAAAAGCAGGTGCTAAAAGAGT
AATTATTTCCGCACCCGCTAAAGAAAAAACTATCAGGACAGTTGTTTACAATGTTAATCACAAAACCATTAGTA
GCGATGATAAGATCATCTCAGCAGCTAGCTGTACTACTAACTGTTTAGCACCATTAGTTCATGTACTTGAAAAG
AACTTTGGGATTGTTTATGGAACGATG

>*Mycoplasma gallisepticum* AE015450

TAACGACTTAACTGATGCTAAGACACTTGCTTATTTACTAAAATATGATACAGCTCACGGTAAGTT---
GAGTCACAACGTGAGT------------TCAACTGATAGTGAGATCGTAGTTGGT------------------
AAACAAAAAATTAAAGTTTACAGTGAAAAAGATCCTACACAAATTCCATGAAAGAAACACAAAGTTGATTTAG
TAGTTGAATCAACTGGTCGTTTTTTAACTCAAGAAGCTGCTAGTGCACACCTTAAAGGTGGTGCTAAGAAAGT
AGTTTTATCAGCTCCAGCTAAAGAAAAGAACGTGAAAACTGTAGTTTACAACGTGAACCACACTGAAATTAAA
CCAGATGATACTGTAATTTCAGCTGCTAGTTGTACAACTAACTGTTTAGCTCCACTTGTTAAAGTGTTAGAAGA
CAAGTTCGGAATTAAAGTTGGTTATATG

>*Mycoplasma arthritidis* CP001047

TAATGATTTAACAGATGCCAAAACTTTAGCACATTTACTAAAATACGACACAGCACACGGAAAAAT---
GAATGCAGAAATCTCA------------CACACCGAAAATTCAATCATTGTTGAC------------------
GGTGTGGAATATCCTGTTTATGCTGAAAAAGATCCTGCAATGCTACCTTGAAAAAAACTTGGTGTTGATATTGT
AGTTGAAGGAACTGGTCGTTTCGTTACCGTTGAAGGTTCACAAAAACACATTGAAGCAGGAGCTAAAAAAGT
TTTAATTACCGCTCCTTCAAAAACTGATAGCGTTAAAACAATAGTTTATTCAGTAAATGAAAATACTTTAAATAA
AGACGATGTTATTGTTTCAGCAGCATCATGTACAACTAACTCTTTAGCACCAGTTATGAATGTCTTAGAAAATG
AATTTGGTGTAGTTAAAGGATATATG

>*Mycoplasma hominis* FP236530

TAATGATTTAACAGATGCAGCTACATTAGCTCACTTATTGAAATACGATACAGCTCATGGAAAAAT---
GAATGAAAATATTTGA------------CACACAGAAAATTCTATTGTTGTAAAT------------------

GGCAAAGAATATCGTGTTTATAGTGAAAAAGATCCATTAAACCTACCTTGAAAAGAACTAAACGTAGATATAG
TTATTGAAGGAACTGGAAGATATGTAACAAAAGAAGGTGCTGAATTACATATTCAAGCAGGTGCTAAAAAGG
TGTTTATTACTGCTCCAGCTAAAAGCGAAGGTGTTAAAACAGTTGTTTATTCAGTAAACGAAGATATCATTACG
CCAGAAGATAAAATTTTATCAGGCGCTTCATGTACTACTAACTGTTTAGCTCCTATTGCCAACGTATTGGAAAA
AAACTTTGGTATTGAAAAAGGATTTATG

>*Mycoplasma fastidiosum* KF151045

TAATGATTTAACTGACGCAAAAACTTTAGCTCATTTATTGAAGTATGATACAGCTCATGGTGCATTGCCAAATT
TTGTTACTGTA------------AATGATAAAAATAATATTTCAATAGAT------------------
GGTAAAGAATTAAAGGTTTTTGCTGAAAAAGATCCATCTAAATTACCTTGAAAAGATTTAGGAATTGATTTAGT
AGTTGAATCAACTGGATTATTTTTAACAAAAGAAAAAGCACAATTACATATTGATGCAGGCGCAAGAAAAGTT
TTATTGTCAGCTCCAGCCAAAGAAAAAAATATTAAAACTGTTGTGTTTAATGTGAATCATGAAATTATTAATTC
AGATGATCAAATAATTTCTGCTGCTTCTTGTACAACTAACTCATTAGCACCTGTTGTTAAAGTTTTAGAAGATAA
TTTTAAGATTGTTGCAGGAACAATG

>*Acholeplasma laidlawii* CP000896

CAACGATTTAACTGATACAGAAACTCTTGCTTACCTTTTAAAGTATGATACAGCTCAAGGTCCTTTCAAAGGTC
ATGAAGTATCA------------ACAAACGGAGATAAATTAGTAGTTGAT------------------
GGTAAATCAATCACTGTTTACGCTCAAAAAGATCCATCTGAGTTACCATGGGGTACACTTGGTGTTGACGTAG
TATTAGAGTCAACAGGATTATTCACATCAGCTGAAAAAGCTGGTTTACACATCAAAGCGGGTGCTAAGAAAGT
TGTAGTTTCTGCACCAGCAACAGGTGAAGGCGTTAAAACTGTTGTTTACAACGTAAACGATGACATCTTAGAT
GGAACTGAAACAATCGTTTCAGCTGCATCATGTACAACTAATGCTTTAGCTCCTTTAGCAAAAGTTTTAGATGA
CAACTTTGGTATCGTTAAAGGTTTCATG

>*Mycoplasma agalactiae* str. 5632 FP671135

TAATGACTTAACTGAACCAAAAATGCTTGCACACTTATTAAAATATGACACTGCTTTTGGCCCATT---
AAAGGCTGATGTAGTT------------GTTAAGGATGATGCTTTTATTGTAAAT------------------
GGCAAAGAAATCAAGGTATTTTCTGAAAAAGATCCTGCCTTGCTGCCTTGAAAAGAACTTAATATTGATATTGT
TTTAGAATGTACAGGTTTTTTTGTTAAAAAAGATCTTGCTCACAAACACATAGAGGCTGGTGCTAAGAAAGTTA
TTATTTCAGCTCCTGCTGGTAA---
AGACGTAAAAACTGTTGTTTATGGTGTAAACCATGAAATTCTATCTGCTGATGATGAAATTATCTCAGGGGCTT
CATGTACAACAAACTGTTTAGCTCCTGTAGTTAAGGTTTTAGTAGATAACTTTGGTATTGAAAATGGATTCATG

>*Mycoplasma agalactiae* str. PG2

TAATGACTTAACTGAACCAAAAATGCTTGCGCACTTATTAAAATATGACACTGCTTTTGGCCCATT---
AAAGGCTGATGTAGTT------------GTTAAGGATGATGCTTTTATTGTAAAT------------------
GGCAAAGAAATCAAGGTATTTTCTGAAAAAGATCCTGCCTTACTGCCTTGAAAAGAACTTAATATTGATATTGT
TTTAGAATGTACAGGTTTTTTTGTTAAAAAAGATCTTGCTCACAAACACATAGATGCTGGTGCTAAGAAAGTTA
TTATTTCAGCTCCTGCTGGTAA---
AGATGTAAAAACTGTTGTTTATGGTGTAAACCATGAAATTCTATCTGCTGATGATGAAATTATCTCAGGTGCTT
CATGTACAACAAACTGTTTAGCTCCTGTAGTTAAGGTTTTAGTAGATAACTTTGGTATTGAAAATGGATTCATG

>*Mycoplasma crocodyli* CP001991

TAATGATTTAACAGACCCAGTTACACTAGCTCATTTATTAAAATACGATACAGCATTTGGAACACT---
AAAAGTTGATGTTGAA------------GCTAAAGAAAATGCTATCGTAGTTAAC------------------
GGAAAAGAAATTAAAGTTTTTGCAGAAAAAGATCCAGAAGCTTTACCATGAAAAGCTCTTGACATTGACCTTG
TTATTGAATGTACAGGTTTCTTTGTTAAAAGAGAAGGAGCAGGAAAACACTTAAAAGCAGGAGCTAAAAAAG
TTGTTGTTTCTGCACCTGCAGGAAG---
TGATGTTAAAACAATCGTTTATAACGTTAACCACAAAACACTTAATGTTAATGATGACATTATTTCTGGTGCTTC
ATGTACAACAAACTGTTTAGCACCAGTAGTTAAAGTTTTAGTTGATAACTTTGGTTTAGAATCAGGATTTATG

>*Mycoplasma felis* KF151046

AAATGACTTAACAGATGCTAAAACATTAGCTCATTTATTAAAATTCGATACTGCTTTTGGTCAATT---
AAAAGCAGAAGTATCA------------GCAAAAGATGGTGCTATTGTAGTAAAT------------------
GGAAAAGAAATCAAAGTTTTTGCAGAAAGAGATCCAGAAAACTTACCATGAGGTCAATTAGGAATTGACTTA
GTTGTTGAATCAACAGGATTCTTTACAAAAAGAGAAGGTGCTGAAAAACATTTAAAAGCAGGTGCTAAAAAA
GTTGTTGTTTCTGCTCCATCAGACAA---
AGATGTTAAAACAATCGTCTACAATGTAAACCACGATGTTTTAACAAAAGAAGATGTTTTAATTTCAGCAGCAT
CATGTACAACAAACTCATTAGCTCCAATGGTAAAAGTATTAGTTGATAATTTTGGTTTAAAATCAGGATACATG

>*Mycoplasma synoviae* AE017245

TAATGATTTAACTGATGCTAAAACTCTAGCTCACCTGCTAAAATACGATACAGCTTTCAAAAAATT---
ACAATTTTCAGTAGAA------------GAAAAAGATAGCTCACTATGAGTTAAT------------------
GGAAAAGAAATTAAAGTTTTTGCTGAAAAAGATCCATCAAATTTACCATGAAAAGATCTAGGAGTAGATTTAG
TAGTTGAATCAACTGGATTTTTCACTAAAAAAGACCTTGCATCTAAGCATTTAGAAGCCGGAGCTAAAAAAGT
TTTAATTTCAGCGCCAGCAGGAAG---
CGATCTTCCTACTGTGGTTTATAACGTAAACCATAAAACTCTAAAAAGTAGTGATACCGTGATTTCTGCTGCAT
CATGTACTACAAACTGCCTTGCGCCTGTGGTAAAAGTTTTAGTTGAAGAATTCGGTCTAAAATCAGGATATATG

>*Clostridium perfringens* BA000016

CAACGACTTAACTGATGCTAAGACTTTAGCACACTTATTCAAATACGATTCAGCACAAGGAAGATT---
CAATGGTGAAATAGAA------------GTTAAAGAAGGAGCTTTCGTAGTTAAC------------------
GGAAAAGAAATCAAAGTAACTGCTAAAAGCAACCCTGCTGAATTACCATGGGGAGAATTAGGAGTAGACGTA
GTATTAGAGTGTACTGGATTCTTCGCATCAAAAGAGAAAGCTTCAGCTCACTTAACTGCTGGTGCTAAAAAAG
TTGTTATCTCAGCTCCTGCTGGAAA---
CGACCTACCAACAGTTGTTTACAACGTAAACCACGATATATTAGATGGAAGCGAAGATGTTATCTCAGGTGCT
TCATGTACTACAAACTGCTTAGCTCCAATGGCTAAAGCTTTAAATGATAACTTCGGATTAAACAAAGGTTTCAT
G

>*Mesoplasma florum* AE017263

TAACGATTTAACAGACACAAAAACATTAGCTTACTTATTAGAATTTGACTCAGCACAAGGAAAATTCCAAGAA
GGAAAAATTTCA------------TACACAGATAACTCAATTATCGTTAAT------------------
GGAAAAGAAATTAAAATCTTTGCTGAAAGAAATGCAGCTGATTTACCTTGAGGTAAATTAGGAATCGATTTAG
TAATCGAATCAACAGGATTCTACACAGATAAAGAAAAAGCATCAGCACACTTAACAGCTGGAGCAAAAAAAG
TTATTATTTCAGCACCTGCTACAGG---
AGAAATGAAAACAATCGTTTATGGTGTAAACCACAAAAACTTATCAGCAGAAGACGTAATTATTTCAGGAGCT

TCATGTACAACAAACTGTTTATCACCTGTTGCTAAAATCATGGATGAAAAATTTGGAATCGTTAAAGGAAAAAT
G

>*Mycoplasma capricolum* CP000123

TAATGATTTAACTGACACTAAAACACTAGCATATTTACTTGAATTTGACACAGCCCAAGGAATTTTTTGCGAAG
ATGAAATTTCA------------TATACTGATAATTCAATTATAGTTAAA------------------
GGAAAAGAAGTTAAAGTATTTGCTGAAAAAGATGCTGCTAACTTACCATGATCTGATTTAAAAATTGATTTAG
TTGTTGAGTCAACTGGATTTTATACAGATAAAGAAAAAGCTTCAGCTCATATTAAAGCAGGTGCAAAAAAAGT
TATTATTTCAGCACCAGCAACTGG---
AGATTTAAAAACTATTGTTTATGGGGTTAACCACAAATCATTAACAAGTGATGATGTAATTATTTCTGGAGCTT
CATGTACAACTAATTGTTTAACACCATTTACTAAAGCTTTAGATGACACATTTACTATTAAAAAAGGTTTTATG

>*Mycoplasma mycoides* Small Colony BX293980

TAATGATCTAACTGACACTAAAACATTAGCTTATTTATTAGAATTTGATACAGCTCAAGGAATCTTTTGTGAAG
GTGAAATCTCA------------CATACAGATAATTCAATTATAATTAAA------------------
GGAAAAGAAGTAAAAGTATTTGCTGAAAAAGATGCTTCTAATTTACCTTGAAGTGAATTAAAAGTTGATTTAG
TAATTGAATTAACTGGATTTTATACAGATAAAGAAAAAGCTTCAGCTCACATTAAAGCAGGAGCTAAAAAAGT
GGTTATTTCAGCTCCAGCAACTGG---
AGATTTAAAAACTGTTGTTTATGGAGTTAACCACAAATCATTAACTAGTGATGATGTAATTATTTCTGGAGCAT
CATGTACAACTAACTGTTTAACACCATTTACTAAGGCTTTAGATGATGCATTTACTATTAAAAAAGGATTTATG

>*Mycoplasma mobile* AE017308

TAATGACTTAACAGATGCTAATACATTAGCACATTTATTTAAATATGATTCTATTTATGGAAATTT---
TAATGGGTCAATAAAAGTT------GATGAAAAAGCTGAATCATTAATTATTAAT------------------
GGTCACAAAATCAGAATTTTGTCAGAAAGTGATCCTTTAAAATTACCTTGAGGTGATTTAGGAATTGATTTAGT
TATTGAATCAACAGGAAGATTTGCAACTAAAGAACAAGCAAGTCAACATTTAAAATCAGGAGCTAAAAAAGTT
TTAATTTCAGCTCCTGCTAAAGGCGCAGGAATTCCAACAGTGGTTCATAATGTAAATCACCAAATTTTAAATGC
AAGTGATACAATTATTTCTACAGCTTCATGTACAACAAATTCATTAGCACCAGTTGCACATGCAATAAATAAAG
AATTTGGAATAGAATCAGGTTTAATG

>*Mycoplasma penetrans* BA000026

TAATGACTTGACTGATGCTAAAACATTATGTCACTTATTAAAATATGACACGGCTCATAGAACATTTAAAGGGA
AGTTATCATAT------------GATGAAAATAACAATTTAATCATTGAT------------------
GGAAAGAAAATCCCAATCTTAGCTGAAAAAGATCCAGCTAACTTACCTTGAGCAAAATTAGGTGTTGATATTG
TTGTAGAATCTACTGGTAGATTTGTTGATGAAGAAGGTGCATCTAAGCACTTAAAAGCTGGAGCTAAAAAAGT
AATTATCTCTGCTCCTGCTAAAGG---
AAACATTCCTACAGTTGTTTACAACGTAAACCACCAAACACTTAAAGCTACTGACAAAATTGTTTCAGCAGCAT
CATGTACTACAAATGCATTAGCACCAGTTGCAAATGTATTAAGTAAAGAATTTGGTATTAAATGAGGATTCAT
G

>*Mycoplasma conjunctivae* FM864216

CAATGATTTAACAGATGTTAAAACCTTAGCACACCTATTTAAATATGATACAGCTCATGGAAAATT---
TGAAGGTAAAGTTGATTATATTCAAGAAGAAGACAAAAGCTTTTTAGTAATTAAC------------------

GGACACAAAATTTTAATTCTTTCCCAAAGAGATCCAAAAACACTTCCTTGAGCTCAATTAGGAATCGATATTGT
GCTTGAATGTACAGGATTTTTCGCTTCCAAAACAGGAGCACAATTACACCTAGACGCTGGTGCTAAAAAAGTT
GTTATTTCTGCACCTGCAGGAAA---
CGATGTTAAAACTATTGTCTACAATATTAACCACGAAACAATTACCGAAGAGGATACAATCTTATCAGCGGCAT
CATGTACCACAAACGCTTTAGCACCGGTTGTAAATGCACTTGAAAAAGAATTTGGAATTGAAAATGGATATAT
G

>*Mycoplasma hyopneumoniae* str. 7448 AE017244

TAACGACTTAACAGATGCATCAGTTTTAGCTCATTTATTTAAGTATGATTCCGCCCATGGAAAGTT---
TAACGGTGAAGTTGAAGTTTTAAAAGATAACGGGAAAAATTACCTTAAAATTAAA------------------
GGTCAAAAAATTCTTGTTTTATCTGAAAGAGACCCAAAATCCTTGCCTTGAGGTCAACTTGGAATTGATTTGGT
TGTTGAATGTACAGGATTTTTTGCTTCAAAATCAGGAGCTAGTCAACATTTAGAGGCGGGTGCAAAAAAAGTA
ATAATTTCTGCTCCGGCAGGAAA---
TGATGTTAAAACCATTGTTTATAACGTAAATTGTGATACAATTACTGAAGATGATAGAATTTTATCCTCAGCTTC
TTGCACTACAAACGCACTTGCCCCACTTGTAAATGCGCTTGATAAAGAATTTGGGATAAACCACGGATTTATG

>*Mycoplasma hyopneumoniae* str. 232 AE017332

TAACGACTTAACAGATGCATCAGTTTTAGCTCATTTATTTAAGTATGATTCCGCCCATGGAAAGTT---
TAACGGTGAAGTTGAAGTTTTAAAAGATAACGGAAAAAATTACCTTAAAATTAAA------------------
GGTCAAAAAATTCTTGTTTTATCTGAAAGAGACCCAAAGTCCTTGCCTTGAGGTCAGCTTGGAATTGATTTGGT
TGTTGAATGTACAGGATTTTTTGCTTCAAAATCAGGAGCTAGTCAACATTTAGAGGCGGGAGCAAAAAAAGTA
ATAATTTCTGCTCCGGCAGGAAA---
TGATGTTAAAACCATTGTTTATAACGTAAATTGTGATACAATTACTGAAGATGATAGAATTTTATCCTCAGCTTC
TTGCACTACAAACGCACTTGCCCCACTTGTAAATGCGCTTGATAAAGAATTTGGGATAAACCACGGATTTATG

>*Mycoplasma hyopneumoniae* str. J AE017243

TAACGACTTAACAGATGCATCAGTTTTAGCTCATTTATTTAAGTATGATTCCGCCCATGGAAAGTT---
TAACGGTGAAGTTGAAGTTTTAAAAGATAACGGAAAAAATTACCTTAAAATTAAA------------------
GGTCAAAAAATTCTTGTTTTATCTGAAAGAGACCCAAAGTCCTTGCCTTGAGGTCAGCTTGGAATTGATTTGGT
TGTTGAATGTACAGGATTTTTTGCTTCAAAATCAGGAGCTAGTCAACATTTAGAGGCGGGAGCAAAAAAAGTA
ATAATTTCTGCTCCGGCAGGAAA---
TGATGTTAAAACCATTGTTTATAACGTAAATTGTGATACAATTACTGAAGATGATAGAATTTTATCCTCAGCTTC
TTGCACTACAAACGCACTTGCCCCACTTGTAAATGCGCTTGATAAAGAATTTGGGATAGACCACGGATTTATG

>*Mycoplasma pulmonis* AL445563

AAATGACTTAACTCAATCTTCAACTTTAATGCACTTATTAAAATTTGACACAGCATATGGAAGATT---
TAACTCAAAAGTTGAA------------TTAACAGAAAAAGGATTTTCAGTAGAT------------------
TCAAAAGAAGTTCTAGTTTTTGCAGAAAGAGACCCAAGAAACTTACCTTGAGGAAAATTGGAAATTGACCTTG
TTCTAGAGTGTACAGGAATGTTTGCTTCAAAAGAAAAATCTCAAGTTCACCTTGATGCAGGGGCTAAAAGAGT
TCTTATTTCAGCTCCAAGTGGCTC---
AGATGTTAAAACTATTGTTTATGGAGTTAATGACTCTTCTCTTTCTTCAGAGGATAAAATTGTCTCAGCTGCTTC
ATGTACAACAAACTGCTTGGCTCCACTTGTAAATGCGCTTGAAAAAGAATATGGAATTCTAACAGGTTCTATG

>*Candidatus* Phytoplasma australiense AM422018

TAATGATTTATCATCTTTAGAAACTATTTCTTATTTGCTTAAATATGATAGTATACAAAGACCTTATGAAGTTGA
TGCTGTTAGT------------TTTGAAGGAAAAAATTTAATAGTTAAA------------------
GGTGAACAAATTCCTGTTTTTCAAGAAAAAAACCCCCAAGATTTGCCTTGGAAAGAATTAGGGGTTGATATTG
TTTTGGAATGTACAGGTTTTTTCACCGATAAAGAAAAGGCATCCTTACATTTAAAAGCAGGCGCTCGCAAAGTT
TTAATTAGTGCTCCTGCGACAGG---
AGATGTTAAAACTATTGTTTATAACGTTAACGATCATACTTTAAATGAAAATGATATTATTGTTAGTGGAGCAT
CATGTACTACTAACTGTTTAGCGCCAATTGTCAAAATTTTAAATGATAATTTCGGTATTAAACAAGCTTTTATG

>Aster Yellows Witches’ Broom Phytoplasma CP000061

TAATGATTTAGCAACTTTAGAAACTATTTCTTATTTATTAAAATACGACAGCATTCAAAAACCTTACAAATTAGA
CGCTGTTAGT------------TTTGAAGACAACTATTTAGTAGTTGAA------------------
GGACAAAAAATTCCTGTATTTCGAGAAAAAAACCCTCAATGTTTACCTTGGAAAAAACTAGGAGTAGATATTG
TTTTAGAATGTACAGGTTTTTTACTAGCAAAGAAAAAGCATCTTTTCATTTAGAAGCTGGAGCTAAAAAAGTG
TTAATTAGTGCTCCTGCTATTGG---
GGATGTAAAAACAGTTGTTTACAATGTTAATGATCAAATCCTAACCAAAGAAGACGCCATTGTAAGTGGGGCT
TCGTGTACTACTAACTGTTTGGCGCCTGTTGTTAAAGTCTTAAATGATAATTTTGGCATCAACCAAGCTTTTATG

>Onion Yellows Phytoplasma AP006628

CAATGATTTAGCAACTTTAGAAACTATTTCTTATTTATTAAAATACGACAGCATTCAAAAACCTTACAAATTAGA
CGCTGTTAGT------------TTTGAAGACAACTATTTAGTAGTTGAA------------------
GGACAAAAAATTCCTGTGTTTCAAGAAAAAAAATCCTCAAGATTTACCTTGGAAAAAACTAGGAGTAGATATTG
TTTTAGAATGTACAGGTTTTTTACTAGCAAAGAAAAAGCAGCTCTTCATTTAGAAGCTGGAGCTAAAAAAGT
GTTAATTAGTGCTCCTGCTACTGG---
GGATGTAAAAACAGTTGTTTACAATGTTAATGACCAAATACTTACTAAAGAAGATGCCATTGTAAGCGGGGCT
TCTTGCACTACTAACTGTTTGGCGCCCGTTGTTAAAGTCTTAAATGACAACTTCGGCATCAACCAAGCTTTTATG

>*Mycoplasma haemocanis* str. Illinois CP003199

AAATGATTTAACTGATCCGAAGACACTTGCTCACCTTCTTAAATATGATACAGCTCATGGTCCTGTTAAGTGTT
ACGATATCAGT------------
GTTGAAGGTGACAGTATTGTTTTAGTTAATAAGTGTAGCGGGGAAAAGCAGTCTTTTAAAGTTATTTCCGAAA
GAGATCCTAAAGCTTTACCTTGAAAATCTTTGGGTGTAGATTGTGTTCTTGAATGTACTGGACGCTTTACTGAT
AAGGATGCAGCCATGGCTCACGTTGAGGCTGGAGCTAAGAAAGTGGTTATCTCTGCTCCAGCAAAAGG---
AGATCTTAAGACAATCGTTTACAACGTAAACCACAATACTTTAACTTCCTCTGATCAAGTTATTTCCGCTGCTTC
TTGTACCACTAATGCTTTAGCTCCCGTTGTAGATGCTCTTCATAAGAAGTACAAAATTGTTTCTGGTTTTATG

>*Mycoplasma haemofelis* str. Langford1 FR773153

CAACGACTTGACTGATCCTAAGACACTTGCTCACCTTCTTAAATATGATACAGCTCATGGACCTGTTAGATGCT
ATGATATCAGT------------
GTTGAAGGTGACAGTATTGTTTTAGTTAATAAATGTAGTGGAGAAAAACAATCCTTCAAAGTTATTTCTGAAA
GAGATCCTAAAGCTCTTCCTTGGAAGTCTTTAAATGTAGATTGCGTTCTTGAATGTACTGGTCGTTTTACCGAT
AAAGATGCAGCTATGGCTCATGTTGAAGCGGGGGCTAAGAAAGTAGTTATCTCCGCTCCAGCAAAAGG---
TGATTTAAAGACAATCGTTTACAACGTAAACCATGGTACTTTAACTTCTTCTGATCAAGTTATCTCAGCAGCTTC
CTGTACAACTAACGCTTTAGCTCCCGTTGTAGATGCTCTTCACAAGAAGTACAAAATTGTTTCTGGGTTTATG

>*Mycoplasma haemofelis* str. Ohio2 CP002808

TAACGACTTGACTGATCCTAAGACACTTGCTCACCTTCTTAAATATGATACAGCTCATGGACCTGTTAGATGCT
ATGATATCAGT------------
GTTGAAGGTGACAGTATTGTTTTAGTTAATAAATGTAGTGGAGAAAAACAATCCTTTAAAGTTATTTCTGAAA
GAGATCCTAAAGCTCTTCCATGAAAATCTTTAAATGTTGATTGTGTTCTTGAGTGTACTGGTCGTTTTACCGATA
AAGATGCAGCTATGGCTCACGTTGAAGCGGGGGCTAAGAAAGTGGTTATCTCTGCTCCAGCAAAAGG---
TGATTTAAAGACAATCGTTTACAACGTAAATCATGGCACTTTAACTTCTTCTGATCAAGTTATCTCAGCAGCTTC
CTGTACAACTAACGCTTTAGCTCCAGTTGTAGATGCTCTTCACAAGAAGTACAAAATTGTTTCTGGGTTTATG

>*Mycoplasma coccoides* KF151044

TAATGACCTTACAGATACAAAAACTTTAGCTCATCTTTTGAAATATGACACAGCTCAAGGTAAGCTTTGTGGTT
GGGATGTAAGT------------
GTTGACGGTTCTCACCTTGTGGTTACTGAAAAATCTTCCGGTAGAGTTGTTAAATGTTTTGTTTGTTCCGAAAG
AGATCCAAAACTTCTTCCTTGGGGAAAATATGGAATTGATTGTGTAATAGAAAGTACAGGTAGGTTTACAGAT
AAAGATTCTGCTATGGCTCATATTGAAGCAGGAGCAAAAAGGGTTGTTATTTCTGCTCCGGCTAAGGG---
TGACCTTAAGACAATTGTTTATAACGTTAACCATCAAATTCTTTCAGGAGACGACAAAGTTATTTCAGCCGCTT
CTTGTACAACAAACGCTCTAGCTCCTGTTGTTAGTGCAATTCACAACAAGTTTGGGGTTGTTTCGGGATTTATG

>*Mycoplasma haemomuris* KF151047

CAATGACCTAACCGATGCCAAAACCCTTGTTCACCTTCTTAAATATGACTCGGCCCACGGACCACTTTGCTGCT
GCTGGAAG---------------
GTTGAGGTTCAAGAGAACAAGTTCATTCTTAGAAGCAGCGAAAAAACCGTTGAAGTTAAGGTATTTGCAGAA
AGAGATCCCGCTAACCTACCTTGGGGAGATCTTGGAATCGACTGCGTAATCGAAAGTACAGGTAGATTCACCG
ACAAAGACGCAGCTTCCGCCCACATTAAAGCCGGTGCAAGAAAGGTTGTTATCTCGGCTCCGGCTAAAGG---
TGACCTTAAAACCATCGTCTTCAACGTTAACCACAATGTTCTTACAAAAGACGACCAAGTTATCTCCGCAGCTT
CCTGTACAACCAACGCTCTTGCCCCAATCGTAAGCGCACTCCACAATTCCTTCGGAGTGGTTTCGGGCTTCATG

>*Mycoplasma suis* str. Illinois CP002525

TAATGACGTAGTTGACATTAAAGTTCTTACTCATCTTTTGGTTTATGACAGTGCTCAAGGAAAACTAAAAGATT
GAGAAGTAAGT------------
TGTGATTCAGAATACATAAGACTAAAGAATGTAAACACCGGAGAAGTTAGAGAAGTTAGAGTTTTCAACTTCA
ATACTGAAAAGATTTATCACTGAGGTGAACTAGAAATTGATTGTGTTGTTGAATGTTCAGGAAGATTCTTGACT
AAGGAAGCGGTTAAGTGTCACCTTGATGCAGGAGCTCAAAAAGTTCTTATTTCAGCCCCAGCAAAGGA---
TGACACTAAGACAGTTGTTTACAACGTAAACCACACTCAAATTACTAGTTCAGACAATGTTATTTCAGGAGCTT
CATGTACAACTAATGCATTAGCTCCTATCGTAAAAATTATTCACAGAAAATTTGGAATTAATTCTGGATTCATG

>*Mycoplasma suis* str. KI_3806 FQ790233

TAATGACGTAGTTGACATTAAAGTTCTTACTCACCTTTTGGTTTATGACAGTGCTCAAGGAAAACTAAAAGATT
GAGAAGTAAGT------------
TGTGATTCAGAATACATAAGACTAAAGAATGTAAATACTGGAGAAGTTAGAGAAGTTAGAGTTTTCAACTTCA
ATACTGAAAAGATTTATCACTGAGGTGAATTAGAAATTGATTGTGTTGTTGAATGTTCAGGAAGATTCTTAACT
AAGGAAGCAGTTAAGTGTCACCTTGATGCAGGAGCTCAAAAAGTTCTTATTTCAGCTCCTGCAAAGGA---

TGACACTAAGACAGTTGTTTACAACGTAAACCATACTCAAATTACCAGCTCAGACAATGTTATTTCAGGAGCTT
CATGTACAACTAATGCACTAGCTCCTATCGTAAAAATTATTCACAGAAAATTTGGAATTAATTCTGGATTCATG

>*Candidatus* Mycoplasma haemominutum str. Birmingham1 HE613254

TAATGATATTGTTGATGCATCGGTTTTAGCTCATTTATTAAAGTATGACAGCTCTCAAGGAGTGTTGAAAGATT
GAGAGGTTAAA------------
AGTGATGCAGAAAACATTTACTTAACTCACATTGATAGCGGAAAACAAAAAACAGTGAAGGTCTTTAATTTCT
TGAAAGAAAAGAGCTACCACTGGGGAGAGTTAGAAGTTGATTGCGTGGTGGAATGTTCGGGACGTTTATTAA
CTAAAGATGCAGTGCAATGCCACTTGGATGCAGGAGCTGAGAAAGTATTAATCTCAGCTCCTGCAAAAGATG
ATGCTATAAAAACTATTGTTTTTAACGTAAATCATAATTCGATTAGCACTTCTGATACGGTAATTTCTGGAGCTT
CTTGCACTACCAATGCATTGGCTCCTGTTGTTAAAGTGTTGCATAGAAAATTTGGAGTGCAGTCTGGATTTATG

>*Candidatus* Mycoplasma haemolamae KF151042

AAATGACATTGTTGACTCGAAAGTTCTTGCTCACCTTCTAAAGTACGACAGCTCTCAAGGGGTTCTTAGAGATT
GAGATGTTACC------------
CACGATCAAGATCACATAAGACTAAAGCATATTTCAACAGGAGTTGAAAAGACAGTTAGAGTATTTGACTTTA
GTAAGGAAAAGAGCTATCACTGAGGAGAGTTAGAAATCGACTGTGTCGTTGAATGTTCAGGAAGACTATTAA
CTAGAGATGCTGTTCAATGTCACCTTGATGCTGGAGCTGAAAAGGTTTTGATCTCGGCGCCTTCGAAGGATGA
AAGCGTTAAGACAATTGTGTTTAACGTAAACCACAATTCGATTGCCACTTCAGATAACGTTATCTCGGGAGCAT
CTTGTACGACAAACGCTCTTGCTCCTATTGTTAAGGTACTACACAGAAAGTTTGGAGTTCAATCAGGATTTATG

>*Candidatus* Mycoplasma erythrocervae KF151043

AAATGACATTGTTGACACAACAGTATTAGCTCACCTTCTGAAATATGACAGTTCACAAGGTGTTCTAAAGGATT
GAGAAGTTACT------------
TGTGATCAAGAAAAGATTTATTTGAAAAACTTAAATAGCGGAGCAGAAAAAAGCTTCAAAGTGTACAACTTTA
CCAAAGAAAAGACTTATCACTGAGGAGAACTAGATGTTGATTGTGTAGTTGAATGTTCAGGGAGACTATTAAC
TAGAGATGCGGTTCAATGTCATTTAGACGCTGGAGCAGATAAGGTATTAATTTCAGCTCCAGCTAAGGATGAC
AGTATTAAGACTGTTGTTTTCAATGTAAATCACAACTCTATTGCCACTTCAGATAATGTAATTTCTGGAGCTTCT
TGTACTACAAATGCGCTTGCTCCTGTAGTTAAGGTTCTTCACAGAAAATTTGGAATCCAATCTGGCTTTATG

>*Mycoplasma wenyonii* KF151049

TAATGATATTGTTGAACCAAGTGTATTGGCACACTTACTAAAGTATGACAGCTCACAAGGTGTATTGGTTGATT
GGGAAATTAGT------------
AGTGATCAAGAAAATATTCACTTAAAGAATCTAAAGAGTGGAGTAGAAAAGAGTTTCAAGGTTTACAACTTCA
ATAAGGAAAAGAGTTACCACTGAGGAGAATTAAATGTTGACTGTGTAGTTGAATGTTCTGGTAGGTTACTAAC
TAGAGAGGCAGTTAAGTGTCACTTGGATGCTGGTGCAGATAAGGTATTAATTTCTGCTCCTGCAAAGGATGAC
AGCATTAAGACTGTTGTATACAATGTGAACCACAACTCAATTGCAACTTCAGATAATGTAATTTCTGGAGCTTC
CTGTACTACAAATGCTTTAGCTCCTGTAGTAAAGGTATTACACAGAAAGTTTGGAATTCAATCAGGATTTATG

>*Candidatus* Mycoplasma haemocervae KF151041

TAATGACATTGTTGATCCTAGTGTATTAGCTCACCTACTAAAGTACGACAGCTCACAAGGAGTATTAACAGACT
GAGAAATCACT------------
AGTGATCAAGAAACTATTTACCTTAAGAATCTAAAGAGCGGAACACAAAAGAGTTTTAAGGTTTACAACTTTA

ACAAGGAAAAAAGTTATCACTGAGGAGAACTTGATGTTGATTGTGTAGTTGAATGTTCAGGTAGATTATTAAC
TAGAGAAGCAGTTAGATGTCACTTGGATGCTGGAGCAGACAAGGTATTAATTTCAGCTCCCGCTAAGGATGA
CAGCATCAAGACTGTTGTTTACAACGTTAACCACAACTCAATTGCTACTTCAGATGATGTAATATCAGGAGCTT
CTTGCACAACAAATGCTCTAGCTCCTGTTGTTAAGGTACTTCACAGAAAGTTTGGAATCCAATCTGGTTTCATG

>*Mycoplasma ovis* KF151048

TAATGACATCGTTGAACCTAGTGTATTGGCACACTTGTTAAAGTATGACAGCTCACAAGGAGTATTAACAGAT
TGGGAAGTAACT------------
AGTGATCAAGAAACTATTTATCTTAAGAATTTAAAAGGAGGGGGAACAAAAAGTTTTAAAGTTTACAACTTTA
ACAAAGAAAAGAGTTATCACTGAGGAGAGCTGGATGTTGATTGTGTAGTTGAATGTTCAGGTAGATTATTAAC
CAGAGAAGCAGTGAGATGTCACTTGGATGCTGGTGCAGACAAGGTATTAATTTCAGCTCCAGCTAAGGATGA
CAGCATCAAGACTGTGGTTTTCAATGTTAACCACAATTCCATTGCTACTTCGGATGATGTAATCTCAGGAGCTT
CTTGTACAACAAATGCTTTAGCTCCTGTTGTTAAGGTACTTCACAGAAAGTTTGGAATCCAATCTGGCTTCATG
