# Supplementary material for: Non-ribosomal phylogenetic exploration of Mollicute species: New insights into haemoplasma taxonomy
Source: Infect Genet Evol. 2014 Apr;23(100):99–105. doi: 10.1016/j.meegid.2014.02.001 (PMC3988868; doi:10.1016/j.meegid.2014.02.001)
Supplement: Supplementary figure B — Fig. B. dnaK sequence alignment. [file mmc2.pdf]

Supplementary Fig. B DnaK sequence alignment

509bp of *dnaK* sequence from Mollicute species used in this study, aligned using MAFFT online multiple sequence alignment programme. Sequences are in fasta format.

>*Mycoplasma haemofelis* str. Langford1 FR773153

ATTGGCTATTAGAGGAAATCAAGAAGGAACAC---TCTATAGATCTTTCTTCAG------------------------------------
----
ATAATTTAGCACTTCAAAGACTTAAGGATGCCGCTGAGAAGGCAAAGATAGAGCTTTCTTCCGTTACTCAAAC
TCAAATACTCCTTCCGTTTCTTTCTATGGTGGGTGGA---
CAACCACTTAACATAGATAAAGTTGTTACTCGTGTTCAGTTTGAATCTTTAACTAAGCATTTAATTGAGAAGAC
TAGGAAGCCCTTCTTGGATGCTTTGAAGGAATCTAAGTTATCTGCTTCCGATATAGATCAAATTCTGTTGGTGG
GTGGTTCTACCCGTATGCCTGCCGTTCAGGAGTTGGTTAAG---
AGCCTTTCCGGGAAGACACCTAACTTGTCTATTAATCCCGATGAAGTTGTTGCTTTAGGTGCTTCCGTTCAAGG
TGCTATTCTTGCTGGGGATATTAA

>*Mycoplasma haemofelis* str. 7415 HM594280

ATTGGCTATTAGAGGAAATCAAGAAGGAACAC---TCTATAGATCTTTCTTCAG------------------------------------
----
ATAATTTAGCACTTCAAAGACTTAAGGATGCCGCTGAGAAGGCAAAGATAGAGCTTTCTTCCGTTACTCAAAC
TCAAATACTCCTTCCGTTTCTTTCTATGGTGGGTGGA---
CAACCACTTAACATAGATAAAGTTGTTACTCGTGTTCAGTTTGAATCTTTAACTAAGCATTTAATTGAGAAGAC
TAGGAAGCCCTTCTTGGATGCTTTGAAGGAATCTAAGTTATCTGCTTCCGATATAGATCAAATTCTGTTGGTGG
GTGGTTCTACCCGTATGCCTGCCGTTCAGGAGTTGGTTAAG---
AGCCTTTCCGGGAAGACACCTAACTTGTCTATTAATCCCGATGAAGTTGTTGCTTTAGGTGCTTCCGTTCAAGG
TGCTATTCTTGCTGGGGATATTAA

>*Mycoplasma haemofelis* str. 1008 HM594283

ATTGGCTATTAGAGGAAATCAAGAAGGAACAC---TCTATAGATCTTTCTTCAG------------------------------------
----
ATAATTTAGCACTTCAAAGACTTAAGGATGCCGCTGAGAAGGCAAAGATAGAGCTTTCTTCTGTTACTCAAACT
CAAATACTCCTTCCGTTCCTTTCTATGGTGGGTGGA---
CAACCACTTAACATAGATAAAGTTGTTACTCGTGTTCAGTTTGAATCTTTAACTAAGCATTTAATTGAGAAAACT
AGAAAGCCCTTCTTGGATGCTTTGAAGGAATCTAAGTTATCTGCTTCTGATATAGATCAAATTCTGTTGGTGGG
TGGTTCTACCCGTATGCCTGCCGTTCAGGAGTTGGTTAAG---
AGCCTTTCTGGGAAGACACCTAACTTGTCTATTAATCCCGATGAAGTTGTTGCTTTAGGTGCTTCCGTTCAAGG
TGCTATTCTTGCTGGGGATATTAA

>*Mycoplasma haemofelis* str. Dalia HM594281

ATTGGCTATTAGAGGAAATCAAGAAGGAACAC---TCTATAGATCTTTCTTCAG------------------------------------
----
ATAATTTAGCACTTCAAAGACTTAAGGATGCCGCTGAGAAGGCAAAGATAGAGCTTTCTTCTGTTACTCAAACT
CAAATACTCCTTCCGTTCCTTTCTATGGTGGGTGGA---
CAACCACTTAACATAGATAAAGTTGTTACTCGTGTTCAGTTTGAATCTTTAACTAAGCATTTAATTGAGAAAACT

AGAAAGCCCTTCTTGGATGCTTTGAAGGAATCTAAGTTATCTGCTTCTGATATAGATCAAATTCTGTTGGTGGG
TGGTTCTACCCGTATGCCTGCCGTTCAGGAGTTGGTTAAG---
AGCCTTTCTGGGAAGACACCTAACTTGTCTATTAATCCCGATGAAGTTGTTGCTTTAGGTGCTTCCGTTCAAGG
TGCTATTCTTGCTGGGGATATTAA

>*Mycoplasma haemocanis* str. Illinois CP003199

ATTGGCTATTGGACGAGATTAAAAAGGAACAT---TCTATAGATTTATCTTCCG---------------------------------------
----

ACAGTTTGGCTCTTCAGAGATTGAAAGATGCCGCTGAGAAGGCAAAGATAGAACTTTCTTCTGTTACCCAAAC
ACAAATACTTCTTCCATTCCTTTCTATGGTAGGAGGG---
CAGCCATTGAATATAGATAAGGTAGTTACTCGTGTTCAGTTTGAATCTTTGACTAAACATTTGATTGAGAGAAC
TAGAAAACCATTTTTGGATGCTTTACAAGAGTCTAAATTGTCTGCTTCCGATATAGATCAGGTCTTGTTGGTGG
GTGGTTCTACCCGTATGCCTGCCGTGCAAGAGTTGGTTAAG---
AGCCTTTCTGGAAAAACTCCTAACTTGTCTATTAATCCGGATGAAGTGGTAGCTTTAGGTGCTTCTGTTCAAGG
TGCTATTCTAGCTGGAGATATTAA

>*Mycoplasma haemofelis* str. Ohio2 CP002808

ATTGGCTATTAGAGGAAATCAAGAAGGAACAC---
TCTATAGATCTTTCTTCAGCTCAAACTCAAATACTCCTTCCGTTCCTTTCTATGGTGGGTGGACAACCACTATAA
TTTAGCACTCCAAAGACTTAAGGATGCCGCTGAGAAGGCAAAGATAGAGCTTTCTTCTGTTA---------------------
----

TAACATAGATAAAGTTGTTACTCGTGTTCAGTTTGAATCTTTAACTAAGCATTTAATTGAGAAAACTAGAAAGC
CCTTCTTGGATGCTTTGAAGGAATCTAAGTTATCTGCTTCCGATATAGATCAAATTCTGTTGGTGGGTGGTTCT
ACCCGTATGCCTGCCGTTCAGGAGTTGGTTAAG---
AGTCTTTCTGGGAAGACACCTAACTTGTCTATTAATCCCGATGAAGTTGTTGCTTTAGGTGCTTCTGTTCAAGG
TGCTATTCTTGCTGGGGATATTAA

>*Candidatus* Mycoplasma haemohominis KF151052

GTTGACTACTTGATGAGATTAATAAAGAACAT---GGAGTTGATTTGAGTAAAG-------------------------------------
----

ATAAGTTGGCTCTTCAAAGAATTAAAGATGCTGCTGAGAAAGCAAAAATTGAGTTGTCTTCAGTTACACAAAC
CCAAATTCTTCTTCCTTTCATATCTATGGTTGGAGGG---
CAACCTTTAAACGTTGATAAGACCGTTACAAGAGTTCAGTTTGAATCTTTGACTAGAAATCTTCTGGATAGAAC
AAGAAAACCGTTTGAGGATGCTCTTAAAGAATCTAATTTAAGTGCGGGCGATATTGATCAAGTATTGTTGGTT
GGAGGCTCTACAAAGATGCCTGCTGTTCAAGAGTTGGTTAAA---
TCTTTATCTGGAAAAAATCCTAACTTGTCTATTAACCCTGATGAGGTAGTTGCTTTAGGAGCATCTGTTCAAGG
TGCTATTTTGGCTGGTGATATTAA

>*Mycoplasma haemomuris* KF151057

ACTGGATCGTTAAAGARGTTCAAAGAGAACAC---GGTGTTGACCTTTACTCKG--------------------------------------
----

ACAACCTGGCTCTTCAAAGGGTTAAGGACGCTGCTGAGAAGGCTAAGATYGAACTTTCCTCCGTGACCCAATC
CCAAATCCTTCTTCCGTTCATCTCCATGGCGGGTGGA---
TCTCCTCTTAACGTTGAGAAAACACTTACAAGGGTTGAGTTCGAATCCCTAACCAAACACCTTCTTGAGAAGAC

AAGACAACCTTTCCTTGACGCYCTKAAAGARGCTAAACTTGAGTCCAAGGACGTTGACCAAATCCTTCTKGTTG
GTGGTTCCACCAAGATGCCATGTGTCCAAAAACTTGTTAGG---
GATCTGTCCGGCAAAAACCCAAACCTATCCATCAACCCAGACGAGGTTGTTGCCCTAGGTGCTTCGGTTCAAG
GTGCTATCCTTGCGGGTGACATCAA

>*Mycoplasma suis* str. Illinois CP002525

AGTGACTTTTGGAATCAATCCAAAAGGAACAT---AGTGTAGACCTATCTAAAG--

ATAACTTAGTAATGCAAAGACTAAAGGAAGCTGCTGAAAAAGCAAAGATTGAACTTTCTTCAGTTCAACAAAC
ACAAATTATGCTTCCTTTCCTTTCAATGGTTCGTGGA---
GAACCATTGAATGTTGATTTCTCATTAACTAGAGAACAATTCCAACTATTTACTAAGGATTTACTAGAAAGAAC
AATAGCACCTGTTAAGGATGCTATTGCAGAATCTAAGTTATCACTTTCAGACATAAATGAAGTTCTACTAGTAG
GTGGTTCTACTAGAATGCCTGCAGTACAAGAACTAGTAGAA---
AAACTAACTGGAAAGAAACCTAATTTGTCTATTAATCCAGATGAAGTAGTAGCTCTAGGAGCTTCTGTTCAAG
CTGGAATTCTAGCAGGAGATATCAA

>*Mycoplasma suis* str. KI_3806 FQ790233

AGTGACTTTTGGAATCAATCCAAAAGGAACAT---AGTGTAGATCTATCTAAAG--

ATAACTTAGTAATGCAAAGATTAAAAGAAGCTGCTGAAAAGGCAAAGATTGAACTTTCTTCAGTTCAACAAAC
ACAAATTATGCTTCCTTTCCTTTCAATGGTTCGTGGA---
GAACCATTGAATGTTGATTTCTCATTAACTAGAGAACAATTCCAACTATTTACTAAGGACTTACTAGAAAGAAC
AATAGCACCTGTTAAGGATGCTATTGCAGAATCTAAATTATCACTTTCAGATATAAATGAAGTTCTACTAGTAG
GTGGTTCTACTAGAATGCCTGCAGTACAAGAACTAGTAGAA---
AAATTAACTGGAAAGAAACCTAACTTGTCTATTAACCCAGATGAAGTAGTAGCTCTAGGAGCTTCTGTTCAAG
CTGGAATTCTAGCAGGAGATATTAA

>*Candidatus* Mycoplasma haemominutum str. Birmingham1 HE613254

ACTGATTACTTGAAACTATTAAAAAAGAACAT---GGAGCTGATCTCTCAAAGG--

ATAATCTTGTGTTACAAAGATTAAAAGAAGCAGCAGAAAAAGCAAAAATAGAACTTTCTTCAGTGCAACAAAC
TCAAATTATGTTGCCCTTCCTAACTATGATTGGAGGA---
GAGCCTCTAAATGTAGACTTAACTCTTTCTAGAGCTCAATTTGAATTACTAACAAAAGATTTACTAGATAGAAC
AGTAAGACCTGTAGAAGATGCTGTCAAAGAATCTCAACTGAAATTAAGTGATATAGATCAAATACTTCTAGTA
GGAGGCTCTACTAGGATGCCAGCAGTGCAAGCACTTGTAGAA---
AAATTAACTGGAAAGAAACCTAATCTTTCAATAAATCCTGATGAGGTAGTAGCATTGGGAGCAGCTGTTCAAG
CAGGAGTACTAGCAGGAGATGTAAA

>*Candidatus* Mycoplasma haemominutum str. Cicuta HM59428

ATTGGTTGTTAGAAACTATAAAAAAAGAACAT---GGTGCAGATTTAGCTAGAG--

ACAATTTAGTTCTTCAGAGATTAAAGGAAGCGGCAGAAAAAGCAAAGATAGAACTCTCTTCAGTTCAACAAAC
TCAAATTATGCTTCCCTTCTTAACTATGATAGGGGGA---
GAGCCTCTGAACATCGACTTGACACTGTCCAGAGGTCAGTTTGAATTATTAACTAAAGATTTGTTAGACAGAA

CTGTGCGACCAGTAGAGGATGCAGTTAAAGAATCTCAATTGCAATTAAGTGATATAGATCAAGTTTTGTTAGT
TGGAGGTTCCACTAGAATGCCTGCTGTTCAAGCTTTGGTAGAG---
CAATTAACTAATAAGAAACCTAATTTATCTATCAATCCCGATGAAGTAGTAGCTTTAGGTGCTGCCGTTCAAGC
CGGAGTGTTAGCAGGAGATGTAAA

>*Candidatus* Mycoplasma erythrocervae KF151050

CTTGATTGTTAGATAGCATAAAGAAAGAACAT---GATGTGGATCTATCTAAAG------------------------------------
----
ATAATTTGGTAATGCAAAGATTGAAGGAATCTGCTGAAAAAGCCAAGATTGAACTTTCCTCAGTTCAACAAAC
TCAAATTATGTTACCTTTCTTAACAATGGTTAAAGGA---
GAGCCCTTGAATGTTGATTTAACTCTTTCCAGATCTCAATTTGAGCTTTTAACAAAAGATCTGTTAGATAGAACT
GAAAAGCCAGTTTTGGATGCTATTGCAGAGTCAAAAGTTGAGTTAAACAAGATCGATGAAGTTCTTTTGGTAG
GAGGTTCTACTAGAATGCCAGCTGTTCAACAATTAGTAGAA---
AGATTAACTAATAAGAAACCTAATCTTTCTATTAATCCTGATGAAGTGGTAGCTTTGGGAGCTTCTGTTCAAGC
TGGTATTTTGGCTGGAGATATTAA

>*Mycoplasma wenyonii* KF151059

ATTGATTACTTGAAACTATCAAGAAGGAAAAC---AATGTAGATCTTTCAAAAG-------------------------------------
----
ATAACTTAGTAATGCAAAGATTGAAAGAGTCTGCAGAAAAGGCAAAGATTGAACTTTCTTCAGTTCAACAAAC
TCAAATTATGTTGCCTTTCTTAACAATGATTAATGGA---
GAGCCTCTAAATGTTGACTTAACTCTCTCTAGATCACAATTTGAGTTATTAACTAAAGATTTACTAGATAGAACT
GAAAAGCCTGTTCTGGATGCAATTAGAGAATCAAAGATTGAGTTAAACCAAATTGATGAAGTTCTTTTGGTTG
GGGGTTCAACCAGAATGCCAGCAGTTCAAGGATTAGTTGAA---
AGATTAACTAAGAAGAAACCAAACCTATCTATTAATCCTGATGAGGTTGTAGCCCTAGGAGCTTCAGTTCAAG
CTGGTATCTTAGCTGGAGATATTAA

>*Candidatus* Mycoplasma haemocervae KF151051

GTTGATTAATTGACACTATCAAAAAAGAACAT---GGTGTAGACGTATCTAAAG------------------------------------
----
ATAATTTGGTATTGCAAAGATTGAAAGAAGCTTCAGAAAAAGCAAAGATTGAATTATCCTCAGTTCAACAAAC
TCAAATCATGTTGCCTTTCCTAACAATGGTTAATGGA---
GAACCTTTGAATGTTGACTTAACTCTTTCAAGATCTCAATTTGAACTTCTTACAAAAGATCTATTAGAAAGAACT
GAAAAGCCTGTTTTGGACGCAATTAAGGAATCAAAGATTGAACTAAATCAAATAGATGAAATTCTTTTGGTGG
GTGGATCTACTAGAATGCCTGCTGTTCAAGAATTGGTTGAA---
AAGTTAACTAAAAAGAAGCCTAATCTATCTATAAACCCAGATGAAGTAGTCGCTCTAGGTGCTTCTGTTCAAGC
TGGAATTTTGGCTGGTGACATAAA

>*Mycoplasma ovis* KF151058

ATTGATTAATTGAAACTATTAGAAAGGAACAT---GGCGTAGATCTTTCTAAAG-------------------------------------
----
ATAATTTGGTAATGCAAAGGTTGAAGGAAGCTTCAGAAAAGGCAAAGATTGAATTATCTTCAGTTCAACAAAC
TCAAATAATGTTGCCCTTCTTAACAATGGTTAACGGA---
GAACCTTTAAATGTTGATTTAACTCTTTCAAGATCTCAATTTGAGCTTCTTACAAAAGACTTATTAGATAGAACT

GAAAGACCTGTTCTAGATGCAATTAAAGAATCGAAGATTGAATTAAAGCAAATAGATGAAGTTCTTTTGGTTG
GCGGCTCTACTAGAATGCCAGCTGTTCAAGCATTAGTTGAA---
AAGTTAACTGGAAAAAAACCTAATTTATCTATAAATCCGGATGAAGTAGTCGCTTTGGGCGCTTCTGTTCAGG
CTGGAATTTTGGCTGGTGATATAAA

>*Candidatus* Mycoplasma haemolamae KF151053

ACTGATTAAAGGAAACCATTCACAAAGAACAT---GGCGTTGATGTTTCTAAAG-------------------------------------
----

ACAATCTTGTTATGCAACGTCTAAAAGAAGCTGCTGAGAAGGCAAAGATAGAACTTTCGTCTGTACAACAAAC
TCAAATAATGTTGCCTTTCTTGACGATGATTCAGGGA---
GAACCTTTGAACGTTGATCTCTCTATGACTAGAGATCAATTCCAGATGCTCTCAAAAGATTTGCTAGATAGAAC
TGAAAAGCCTGTAAAGGATGCTATGAAAGAATCAGGCTTTGAACTTAAGGATATTGATGAAGTTTTGTTGGTT
GGAGGATCCACCAGAATGCCTGCTGTTCAAGATCTTGTAGAG---
AAGTTGACTAACAAGAAACCTAACCTATCTATCAACCCTGATGAAGTGGTAGCTTTGGGCGCTTCTGTTCAAGC
AGGTATCTTGGCTGGCGAAATTAA

>*Candidatus* Mycoplasma kahaneii KF151054

AGTGAATTCTAGATACAGTACAGAAGGAACAT---GGAGTAGATYTATCCAAGG-------------------------------------
----

ATAAGACTGCTATGCAACGTCTTAAGGATGAATCAGAAAARGCTAAGATCTCTCTTTCTTCAGTACAACAAACA
CAAATCTTGCTTCCATTTATAACTATGGTGCAGGGC---
CAACCTTTAAATGTAGATTTAACCCTAACAAGATCTCATTTTGAGGCAATTACTAAAGATTTATTAGATAGAAC
TGTTAAGCCTGTGAAGGATGCTATTCAGGAGTCTAAGTTGGAGTTGTCTCAGATTGACCAGGTGCTGTTAGTT
GGGGGATCTACTAGAATGCCTGCAGTTCAGGCRTTAGCAGAG---
GAATTAACTAAAAAGAAGCCTAATCTTTCTATCAACCCTGATGAGGTAGTTGCACTTGGMGCTGCTGTACAGG
CAGGGGTTCTGGCTGGAGATATTAA

>*Mycoplasma agalactiae* str. 5632 FP671135

AATGATTAATTGATTTAATCAAAAAAGACTAT---AAAACAGATGTTACAAACA---------------------------------------
----

ATAAAATAGCAATGGCTCGTTTAAAAGCAGCAGCTGAAAAAGCAAAAATTGACTTATCTAGTTCACAACAAGC
TACAATTATGCTTCCATTTTTAGTTATGCAACAAGGCTCAGAACCTATTAGTGTTGAAGCAACCTTAAGAAGAA
GTCAATTTGAAGAAATGACATCACATTTGGTTGAAAGATGTAGAAAACCTATTGAAACAGCTTTAGCTGATGC
TAAAATCAAGATTTCTGATTTAGATGACGTAATTTTAGTTGGTGGCTCAACAAGAATTCCTGCAGTGCAACAAT
TAGTTGAA---
TCGATTTTAAATAGAAAAGCTAACCGTTCAGTTAATCCTGATGAAGTTGTAGCTATGGGTGCCGCTATTCAAG
GTGCTGTTTTAGCTGGCGAAATCGA

>*Mycoplasma agalactiae* str. PG2 CU179680

AATGATTAATTGATTTAATCAAAAAAGACTAT---AAAACAGATGTTACCAACA---------------------------------------
----

ATAAAATGGCAATGGCTCGTTTAAAAGCGGCAGCTGAAAAAGCAAAAATTGACTTATCTAGTTCACAACAAGC
TACAATTATGCTTCCATTTTTAGTTATGCAACAAGGTTCAGAACCTATTAGTGTTGAAGCAACCTTAAGAAGAA
GTCAATTTGAAGAAATGACATCGCATTTAGTTGAAAGATGTAGAAAACCTATTGAAACAGCTTTAGCTGATGC

TAAAATCAAGATTTCTGATTTAGATGACGTAATTTTAGTTGGGGGTTCAACAAGAATTCCTGCAGTGCAACAAT
TAGTTGAA---
TCAATTTTAAATAAAAAAGCCAACCGTTCAGTTAATCCTGATGAAGTTGTAGCAATGGGTGCCGCTATTCAAG
GTGCTGTTTTAGCTGGCGAAATCGA

>*Mycoplasma alligatoris* ADNC01000022

ATTGATTAGTATCAGTAATAAAAAGAGATTAT---AAATTTGATGCTTCAACTG---------------------------------------
----
ACAAAATGGCAATGGCTCGTTTAAAAGAAACAGCAGAAAAAGCAAAAATTGATTTATCAAGCCAAGCAGTTG
CAACAATTAATTTACCATTCTTAGCAGTTACACCTGAA---
GGCCCAATTAACGTTGAAACTGAATTAAAGCGTAGTGAATTTGAAGCTATGACAAGCGCATTATTAGATAGAA
CAAGAAAACCTATTGAAGATGCTTTAAAAGAAGCAAAAATTACCGCAAACGACTTGCATGAAGTTCTTTTAGT
TGGTGGATCAACAAGAATGCCAGCGGTTCAAGATATGGTTAAA---
CGTACATTAGGTAAAGAACCAAATAGATCAATTAACCCTGATGAAGTTGTTTCAATAGGTGCAGCAATTCAAG
GTTCAGTTCTAGCTGGAGATATTGA

>*Mycoplasma crocodylii* CP001991

AATGATTAACAGGTCTTATTAAAAAAGAACAC---AATTTTGATGCATCAACCG--------------------------------------
----
ATAAAATGGCTATGGCTCGTTTAAAAGAAGCTGCAGAAAAAGCAAAAATCGATTTATCAAACCAATCAATTGC
TACAATTAACTTACCTTTCTTAGCGGTTACACCATCA---
GGACCTATTAACGTTGAAGTTGAATTAAAACGTAGTGAATTCGATGCTATGACAGCAGGTTTAGTTGATAGAA
CAAGAAAACCTATTGAAGACGCTTTAAGAGAAGCTAAAATTACCGCAAAAGACTTGCATGAAGTTCTATTGGT
CGGAGGTTCAACAAGAATACCTGCAGTTCAAGACATGGTTAAA---
AGAACTTTAGGAAAAGAACCAAACAGAACAATCAATCCAGATGAAGTTGTTTCGATTGGTGCTGCAATTCAAG
GATCTGTTTTAGCTGGTGATATTGA

>*Mycoplasma conjunctivae* FM864216

ATTGAATGGTAGCTAAAATTAAACAAGAATAC---AACTTTGATGCAACTAAAG--------------------------------------
----
ACAAAATGGCTTTATCTAGATTAAAAGAAGAAGCTGAAAAAGCAAAGATTAACTTATCAAACCAAATGGTTAC
CACAATTACACTACCTTTTTTAGGTATGAATGAAAAT---
GGACCAATTAATGTTGAACTTGAGCTCAAAAGATCTGAATTTGAAAAGCTTAGCGCCCACTTGGTAGACCGTA
CTAGAAAACCAATTGTTGATGCGCTCAAAGAAGCCAAATTAGAAGCTAAAGACCTCGATGAAATTTTATTAGT
TGGAGGTTCAACTCGAATTCCTGCTGTCCAAACAATGATTGAG---
CACACCTTAAATAAAAAGCCAAATCGCTCAATTAATCCAGATGAGGTAGTAGCTATTGGAGCTGCTATTCAAG
GGGGGGTTTTAGCTGGTGAAATCAA

>*Mycoplasma hyopneumoniae* str. 232 AE017332

ATTGACTTGTTAAAAAAATCAAAGAAGTATAT---GATTTTGATCCAAAAAGTG--------------------------------------
----
ATAAAATGGCGCTTACAAGACTTAAAGAAGAGGCTGAAAAAACCAAAATTAATCTTTCAAATCAAAGTGTTTC
TACAGTTTCTCTACCATTTTTAGGAATGGGCAAAAAC---
GGGCCGATTAACGTTGAACTTGAACTTAAAAGATCAGAATTTGAAAAAATGACTGCCCATTTAATCGATAGAA

CTCGCAAACCAATTGTTGATGCTCTAAAACAAGCAAAAATTGAGGCTTCAGATCTTGATGAAGTTCTCCTTGTA
GGTGGATCAACAAGAATGCCAGCTGTTCAGTCAATGATTGAG---
CATACTTTAAATAAAAAGCCAAATCGTTCAATTAATCCTGATGAGGTAGTCGCAATTGGTGCTGCAATTCAAG
GGGGGGTTCTAGCTGGAGAGATCAG

>*Mycoplasma hyopneumoniae* str. 7448 AE017244

ATTGACTTGTTAAAAAAATCAAAGAAGAATAT---GATTTTGATCCAAAAAGTG---------------------------------------
----
ATAAAATGGCGCTTACAAGACTTAAAGAAGAGGCTGAAAAAACCAAAATTAATCTTTCAAATCAAAGTGTTTC
TACAGTTTCTCTACCATTTTTAGGAATGGGCAAAAAC---
GGGCCGATTAACGTTGAACTTGAACTTAAAAGATCAGAATTTGAAAAAATGACTGCCCATTTAATCGATAGAA
CTCGCAAACCAATTGTTGATGCTCTAAAACAAGCAAAAATTGAGGCTTCAGATCTTGATGAAGTTCTCCTTGTA
GGTGGATCAACAAGAATGCCAGCTGTTCAGTCAATGATTGAG---
CATACTTTAAATAAAAAGCCAAATCGTTCAATTAATCCTGATGAAGTAGTCGCAATTGGTGCTGCAATTCAAGG
GGGGGTTCTAGCTGGAGAGATCAG

>*Mycoplasma hyopneumoniae* str. J AE017243

ATTGACTTGTTAAAAAAATCAAAGAAGAATAT---GATTTTGATCCAAAAAGTG---------------------------------------
----
ATAAAATGGCGCTTACAAGACTTAAAGAAGAGGCTGAAAAAACCAAAATTAATCTTTCAAATCAAAGTGTTTC
TACAGTTTCTCTACCATTTTTAGGAATGGGCAAAAAC---
GGGCCGATTAACGTTGAACTTGAACTTAAAAGATCAGAATTTGAAAAAATGACTGCCCATTTAATCGATAGAA
CTCGCAAACCAATTGTTGATGCTCTAAAACAAGCAAAAATTGAGGCTTCAGATCTTGATGAAGTTCTCCTTGTA
GGTGGATCAACAAGAATGCCAGCTGTTCAGTCAATGATTGAG---
CATACTTTAAATAAAAAGCCAAATCGTTCAATTAATCCTGATGAGGTAGTCGCAATTGGTGCTGCAATTCAAG
GGGGGGTTCTAGCTGGAGAGATCAG

> *Mycoplasma pulmonis* AL445563

AATGATTAACTAAAGAAATTAAAAATAGATAT---GATTTTGACCCTTCAAAAG---------------------------------------
----
ACAAAATGGTAATGACAAGACTTAAAGAAGCAGCTGAAAAAGCAAAAATTGACCTTTCAGCACAAATGGTAG
CTCAAATTACTCTTCCTTTCTTGTCAGTTACTTCTAAA---
GGACCTATTAACGTTGATTTAGAACTAAAAAGAAGTGAATTTGAAAAAATGACAACTCATTTAGTTGATAGAA
CTAGAAAACCTATTGAAGATGCTCTAAGAGAGGCAAAAATTAAAGCAAGTGACCTTAGTGAAGTTCTTTTAGT
TGGAGGATCAACTAGAATTCCTGCTGTTCAATCTATGGTAGAG---
CATGTTCTTGGTAAAAAACCAAATCGTTCAATTAACCCTGATGAAGTTGTTGCTATTGGAGCTGCAATTCAAGG
TGGAGTTTTAGCAGGAGATATCAA

>*Mycoplasma mobile* AE017308

AATGATTAATCGGAAAAATTAAATTAGAACAT---AAATATGATGTTTCAAAAG---------------------------------------
----
ATAAAATGGCAATGGCTAGATTAAAAGAAGAAGCAGAAAAAGCAAAAATTAATTTATCTACAACATCTACAAC
ATCAATTAATTTACCCTTTTTAGCAGTAACAGATTCA---
GGACCTATTAATGTTGAAGTCGAATTAAAAAGAAGTGATTTTGAAAAAATGACTCAACATTTAGTTGAAAGAA

CAAGAAAGCCTGTTAGAGATGCTTTAAAAGAAGCAAAATTAAAATCAGAAGATTTACATGAAGTTTTACTTGT
AGGGGGTTCAACAAGAATTCCTGCTGTTCAAGAAATGTTGCAA---
CACGAGTTGAACAAAAAACCAAATCATAGTATTAATCCAGATGAAGTAGTAGCAATTGGTGCTGCTATTCAAG
GGGCTGTTCTTTCTGGAGATATTAA

>*Mycoplasma synoviae* AE017245

AATGATTAACTAAAGAAATAAAAACAAAATAC---AGCTACGATGTATCTAAAG------------------------------------
----

ATAAATACGCTTTAGCTCGTTTAAAAGAAAACGCTGAAAAAGCTAAAATTGATCTATCAAATCAATCAGTTGTG
CAAATTAATATTCCATTTTTAGCAATGTCAGCTAAT---
GGGCCAATCAACGTTGAGCTTTCTCTAAAAAGAAGTGAATTTGAAGCAATGACTTCACATTTATTAGATAGAA
CCAGAAAACCTATCGAAGACGCTCTTAAAGAAGCAAAACTAAGTGCTAATGACATTCACGAAGTGCTTTTAGT
AGGTGGATCTACTAGAATGCCAGCGGTGCAAGATATGGTTAAA---
AGAACTTTAGGAAAAGAACCTAACCGTTCAATTAACCCTGACGAAGTTGTATCTATAGGAGCTGCTATCCAAG
GAGGAGTGCTAGCCGGACATATCGA

>*Mycoplasma arthritidis* CP001047

AATGAATGGTTGAAGAAATTAAAAATAAATAC---AACTATAACCCAGAAACTG------------------------------------
----

AAAAAATGGCAATGGCAAGACTAAAAGAAGAAGCTGAAAGAGCAAAAATTACTCTTTCTGAAAGTATGGTTG
CTAATATTTCTCTACCATTCTTGGCCATGTCACAAACA---
GGACCAATTAATGTCGAATTGGAACTAAAAAGATCGGAGTTTGAAAAAATGACTGACGATCTACTACAAAGA
ACTAAAAAACCACTATTAGATGCATTAAGCGAAGCAAAATTACAAATTAATGATCTAGATGAAGTTTTATTAGT
TGGTGGTTCAACTAGAATGCCCGCTGTTCAAAAATTAGTAGCT---
GATACCATTAATAAAAAACCTAACAATTCTATTAACCCTGATGAAGTAGTTAGTGTTGGAGCCGCTATTCAAGG
AGCTATCTTAGCTGGTGATGTTCA

>*Mycoplasma hominis* FP236530

AATGAATGGTTGAACAAATTAAATCTAAATAC---AATTTCGATCCAACAACTG-------------------------------------
----

ACAAAATGGCAATGGCAAGATTAAAAGAAGAAGCAGAACGTGCAAAAATTACTTTATCAGAACAATTAATTG
CAAACATTTCTCTTCCATTCTTAGCAATGAATGAAAAT---
GGACCAGTTAATGTTGAATTAGAAATTACTCGTGCTACATTTGAATCAATGACTGAACATTTACTACAAAGAAC
AAGAAAACCATTATTAGATGTTTTAAGTGAAGCAAAATTAACATGAAATGATATTAATGAAGTATTACTAGTTG
GTGGTTCAACAAGAATGCCAGCTGTTCAAAAACTAGTAGCA---
GAAGTAACTAATAAAAAACCAAATAATTCAATTAACCCAGATGAAGTAGTTAGCGTTGGAGCTGCTATTCAAG
GTGCAATATTAGCAGGAGAAATTCA

>*Mycoplasma capricolum* CP000123

AATGATTACTAGGTAAAATTAAAGCTGAATAC---AATATTGATTTATCTAAAG--------------------------------------
----

AAAAAATGGCTTTACAAAGATTAAAAGATGAAGCTGAAAAAGCAAAAATTAATTTATCTAGCCAATTAGAAGT
TGAAATTAATTTACCATTTATTGCAATGAATGAAAGT---
GGACCAATTTCTTTTGCAACAACTCTAACAAGAAGTGAATTTAACAAAATTACAAAACATTTAGTTGACTTGAC

TATTCAACCAGTTAAAGATGCTTTAAGTGCTGCTAAAAAAACTCCAAGTGAAATTAATGAAGTTTTATTAGTTG
GTGGGTCAACAAGAATACCTGCTGTTCAAGAATTAGTAAAA---
AGTTTATTAAATAAAGAACCAAATAGATCAATTAATCCAGATGAAGTTGTTGCTATGGGTGCTGCTGTGCAAG
GTGGAGTTTTAGCTGGTGAAGTTAC

>*Mycoplasma mycoides* Small Colony BX293980

AATGATTACTAGGTAAAATTAAAGCTGAATAC---AATATTGATTTATCTAAAG--------------------------------------
----

AAAAAATGGCTTTACAAAGATTAAAAGATGAAGCAGAAAAAGCAAAAATTAATTTATCTAGTCAATTAGAAGT
TGAAATTAATTTACCATTTATTGCAATGAATGAAAGT---
GGACCAATTTCTTTTGCAACAACTCTAACAAGAAGTGAATTTAACAAAATTACAAAACATTTAGTTGATTTAAC
TATTCAACCAGTTAAAGATGCTTTAAGTGCTGCTAAAAAAACTCCAAGTGAAATTAATGAAGTTTTATTAGTAG
GTGGATCAACAAGAATACCAGCTGTTCAAGAATTAGTAAAA---
AGTTTATTAAATAAAGAACCAAATAGATCAATTAATCCAGATGAAGTTGTTGCTATGGGTGCTGCTGTTCAAG
GTGGAGTTTTAGCTGGTGAAGTTAC

>*Mesoplasma florum* AE017263

ACTGATTAATTGAAAAAATTAAAACTGAAAGT---GGTGTTGATTTAAAAAACG-------------------------------------
----

ATAAAATGGCATTACAAAGATTAAAAGATGAAGCTGAAAAAGCAAAAATTAATTTATCAAGTCAATTAGAAGT
TGAAATTAACTTACCATTTATTGCAATGAATGAAAAT---
GGACCAGTTTCATTCTCAACTCAATTTTCAAGAACAGAATTTGACAAAATTACAAAAGATTTAGTTGAAAGAAC
TTCTAAACCAGTAAAAGATGCATTACAAGCAGCTAAATTAAGTGCTAGTGATATTGATGAAGTTTTACTAGTTG
GTGGATCAACAAGAATACCAGCTGTTCAAAAAAATTGTTAAA---
GAATTATTAGGAAAAGAACCAAACCGTTCAATTAACCCAGATGAAGTTGTAGCTATGGGTGCAGCTATCCAAG
GTGGGGTTTTAGCGGGAGATGTTAC

>*Mycoplasma penetrans* BA000026

ACTGAATTGTTGAAGAAGTTAAGAAGAATGAT---AAAGTTGATTTATCAAATG------------------------------------
----

ACAAAATGGCAATGCAAAGATTAAAAGATGCTGCAGAAAAAGCTAAGATTGATCTTTCGGGATTAAAAGAAG
TTGAAATTAGTTTACCTTTTATTGCTATGACTGAAAGT---
GGCCCATTAAATGTAGATCTAAAATTAACAAGAGCTAAATTTGAAGATCTAACAAGAGACTTATTAGAAAGAA
CTATCAAACCAGTTGAAGATGCTTTAAAAGAAGCTAAATTATCAGCAAGTGATATTCATAAAGTTTTATTAGTT
GGTGGTTCTACTAGAATGCCAGCAGTTGAAGAACTAGTTAAA---
TCTAAGCTAGGAAAAAGTCCTGATAAAAACATCAATCCTGATGAAGTAGTTGCTGCAGGTGCTGCTATTCAAG
GTGGTGTATTAATGGGGGATGTTAA

>*Mycoplasma cavipharyngis* KF151055

ATTGATTAATTACTGAAATCCGTAATGAACAC---AGTATTGATTTAACTAAAG--------------------------------------
----

ATAAAATGGCAATGCAACGATTAAAAGATGCTGCAGAAAAAGCCAAAATTGATTTATCAGGTTTAACATCAGT
TCAAATTCTTTTACCATTTTTATCAATGAGTCATGGT---
CAACCATTAAATGTTGATAAAACTTTAACTAGAACTCATTTTGAAAATTTAACTAAAGATTTATTGAACCGAACT

ATTAAACCATTAGAAGACGCTTTAAAAGAAGCAAAAATTGAAACTAGTGAGTTAAACCAAGTTTTATTAGTTG
GTGGTTCAACAAGAATGCCTGCTGTTCAAGCAATGGTTGAA---
AAAATTACTGGTCGCAAACCAAATTTATCAATTAATCCTGATGAAGTAGTAGCATTAGGTGCTGCTGTACAAG
CTGGAGTTTTAAGTGGATCAGTTAA

>*Acholeplasma laidlawii* CP000896

AATTCTTAGCAGCTGAATTTAATAAAGAACAC---GGTATTGATTTACTAAAAG-------------------------------------
----

ATAAAATGGCTTTACAACGTCTACGTGATGCAGCAGAAAAAGCTAAGAAAGAATTATCAGGTGTAACAACAA
GTTCTATTTCTCTACCATTTATTACAATGGGAGCAGCA---
GGTCCGTTACACTTTGAATATAACTTAACGCGTGCTAAATTTAATGAATTGACTAAAGATTTAGTAGATCGTAC
AATGGTATCTGTTAGACGTGCTTTAGCTGATGCTAAATTAACAAACTCTGATATTAATCAAATCTTATTAGTTG
GTGGTTCAACAAGAATTCCAGCTGTTCAAGAACTTGTAACA---
AAAGAATTTGGTAAAGAACCTAATAGATCGGTAAACCCTGATGAAGTTGTAGCAATGGGTGCTGCAATCCAA
GGTGCTGTTTTAGCAGGGGATGTTAA

>*Candidatus* Phytoplasma australiense AM422018

ATTTTTTAGTTAAAGAATTCAAGAAAGATAAC---GGAGTTGATTTATCAAAAG-------------------------------------
----

ATAAAATGGCAATGCAACGTTTAAAAGATGTTGCTGAAAAAGCTAAAAAAGAATTAAGTGGTGTCACTTCTAG
CCAAATTTCTTTACCTTTCTTAACTATGAGCGCGTCA---
GGTCCTTTGCATTTAGAATACAATATGACACGTGCGAAGTTTAATGAATTAACTAAAGATTTAATTGATCGTTG
TTTAGGTCCTGTTAAACGTGCTTTGAGTGATGCTAAATTAACTATTGAAAAAATCGATCAAGTTCTTTTAGTGG
GTGGTTCTACTCGTATTCCTGCTGTTCAAGAATTAGTTAAA---
AAAGAATTACAAAAAACTCCTAATAAAAGTATTAATCCAGATGAAGTAGTTGCAATTGGTGCTGCTATTCAAG
GAGGTATTTTAGCAGGAGATGTGAA

>Aster Yellows Witches’ Broom Phytoplasma CP000061

ATTTCTTAGTGCAAGAATTCAAAAAAGAAAAT---AGTGTTGATCTTTCCAAAG-------------------------------------
----

ATAAAATGGCAATGCAAAGATTAAAAGATGCTGCTGAAAAAGCTAAAAAAGAATTAAGTGGTGTTGCTTCTTC
ACAAATTTCTTTGCCTTTCTTAACAATGAGCGAAGCA---
GGTCCCCTTCATTTAGAATACAATATGACTCGTGCTAAATTCAATGAACTTACTAAAGATTTAATTGATCGTTGT
TTGGCTCCTGTAAAACGTGCTTTAAGTGATGCCAAATTAGATATTAAAAAAATCGATCAAGTGCTTTTAGTGGG
TGGTTCTACTCGTATTCCTGCAGTTCAGGATTTAGTTAAA---
AATGAATTGAATAAAACTCCTAATAAGAGCATTAATCCTGATGAAGTAGTTGCTATTGGAGCTGCTATTCAAG
GAGGAATTCTATCAGGAGAAGTTAA

>Onion Yellows Phytoplasma AP006628

ATTTCTTAGTGCAAGAATTCAAAAAAGAAAAT---AGTGTTGATCTTTCCAAAG-------------------------------------
----

ATAAAATGGCAATGCAAAGATTAAAAGATGCTGCTGAAAAAGCTAAAAAAGAATTAAGTGGTGTTACTTCTTC
ACAAATTTCTTTGCCTTTCTTAACAATGAGCGAAGCA---
GGTCCACTTCATTTAGAATACAATATGACTCGCGCTAAATTCAATGAACTTACTAAAGATTTAATTGATCGTTGT

TTGGCTCCTGTAAAACGTGCTTTAGGTGATGCTAAATTAGATATTGAAAAAATTGATCAAGTGCTTTTAGTGGG
TGGTTCTACTCGTATTCCTGCAGTACAAGATTTAGTTAAA---
AATGAATTGAAAAAAACTCCTAATAAGAGTATTAATCCTGATGAAGTAGTTGGTATTGGAGCTGCTATTCAAG
GAGGAATTCTATCAGGAGACGTTAA

>*Mycoplasma gallisepticum* AE015450

AATGGATCATTGCTGAAATCAAAAAAGATCACCCATCATTAGACCTTAAGTCTG------------------------------------
----

ATAAGATGGCAATGCAAAGATTAAAAGAAGCTGCTGAAAGAGCTAAGATCGAACTATCAGCTCAATTAGAAA
CACTAATCTCATTACCATTCATCGCAGTTACTCCTGAA---
GGTCCAGTAAACGCTGAATTAACTTTATCAAGAGCTAAATTCGAAGAATTAACTAAAGACTTACTAGAAAGAA
CAAGAAACCCAATTGCTGACGTATTAAAAGAAGCTAAGGTTGATCCTAGTCAAGTTGATGAAATTCTTTTAGT
AGGTGGTTCTACAAGAATGCCTGCAGTACAAAAATTAGTTGAATCAATGATTCCTAATAAAGCACCAAACCGT
ACGATTAACCCTGACGAAGTAGTAGCGATCGGTGCTGCTGTACAAGGTGGGGTATTACGTGGGGATGTTAA

>*Mycoplasma fastidiosum* KF151056

ATTGAATAGTTGAAAACATTACTAAAGATCATCCGAATTTAAAAATTCGTGAAG------------------------------------
----

ACAAAATGGCTATGCAACGTTTAAAAGAGGCTGCTGAAAGAGCTAAAATTGAGTTATCTGCTCAATTAGAAGT
TAATGTTTCATTACCATTCATTGCTGTAACTGAATCT---
GGTCCAGTTAACTTTGATATGCAATTATCTAGATCTAAATTTGAACAATTAACTAAGGATTTAGTTGAAAGAAC
AAGAAACCCAATTAAAGACGTATTAACAGAAGCTAAAGTTGATCCTTCTCAAGTTGATGAAATTTTATTAGTTG
GTGGTTCTACAAGAATTCCAGCTGTTCAAGCTTTAGTTGAATCAATGGTTCCTAATAAGAAACCTAACCGTACT
ATTAACCCCGATGAAGTTGTTGCAGTAGGTGCTGCAGTTCAAGGTGGTGTATTACGTGGTGATGTTAA

>*Mycoplasma genitalium* L43967

AATATATCTCAGCCTACATTGCCAAAGAACACCAGGGTTTAAACTTATCAAAAG------------------------------------
----

ATAAGATGGCAATGCAACGGCTTAAAGAAGCAGCTGAACGTGCTAAGATTGAACTTTCCGCTCAACTTGAAAC
GATTATTTCTCTACCATTTTTAACTGTTACCCAAAAA---
GGTCCTGTTAACGTTGAGTTAAAACTAACCCGTGCTAAGTTTGAGGAGTTAACAAAACCACTACTTGAAAGAA
CAAGAAACCCTATTTCAGATGTTATCAAGGAAGCTAAGATTAAACCTGAAGAGATTAATGAAATTCTTTTAGTT
GGTGGTTCTACAAGGATGCCTGCAGTTCAAAAGCTAGTTGAATCAATGGTACCAGGTAAAAAACCAAACCGTT
CTATTAATCCTGATGAAGTTGTTGCTATTGGCGCTGCTATTCAAGGTGGGGTTTTACGTGGTGATGTTAA

>*Mycoplasma pneumoniae* U00089

AGTTCATCTTAGCGCACATTGCCCAAGAACACAATGGGCTTAACTTGTCCAATG------------------------------------
----

ACAAGATGGCTATGCAACGCTTAAAGGAAGCGGCTGAACGTGCTAAGATTGAACTTTCCGCCCAACTAGAAG
CAATTATCTCTTTACCGTTCTTAACGGTTACCGAAAAG---
GGTCCGGTAAACGTTGAACTTAAGCTAACCCGTGCTAAGTTTGAAGAAATTACCAAACAATTACTAGAACGTA
CTCGCAACCCAATTTCGGATGTTTTACGTGAAGCCAAGATTAAACCAGAAGAAATTAATGAAATCTTGTTGGT
GGGTGGATCGACCCGGATGCCAGCAGTGCAAAAACTAGTGGAATCAATGGTACCAGGACACAGTCCAAACCG
CTCAATTAACCCGGATGAGGTGGTAGCCATTGGTGCTGCCATCCAAGGGGGTGTGTTACGCGGTGATGTAAA

>*Ureaplasma parvum* serovar 3 CP000942

ATTGATTATTAAAATCTATTGCTGATGAATTC---AATATTGATTTAAGTAAAA--

ACAAAATGGCTATGCAACGTTTAAAAGATGCTGCTGAAAAAGCTAAAATTGAATTATCAGGTATAAACACAAC
AACTATTTCATTACCTTTTATTGCAATGGATAGTTCTGGTCAGCCAATTAATTTTGAAAAAGAATTAAATCGAGC
GACATTTGATAATCTAACTAAAAATTTAATTGAAAGATTAAAAAAACCTGTTTTAGATGCAATGAAAGAATCTA
AATTATCATTGGTTGATATTGATCAAGTTTTGATGGTAGGTGGTTCAACACGTATGCCTGCAGTACAAAATTTA
GTGAAA---
GAATTAACAGGTAAAGAACCAAACCATTCACTAAATCCTGATGAAGTTGTTGCAATTGGTGCAGCTATTCAAG
GTGGAGTATTAGCAGGAGAAATTGA

>*Ureaplasma urealyticum* serovar 10 CP001184

ATTGATTATTAAAATCAATTGCTGATGAATTT---AATATTGATTTAAGTAAAA--

ACAAAATGGCTATGCAACGTTTAAAAGATGCTGCTGAAAAAGCTAAAATTGAATTATCGGGCGTAAACACAAC
AACTATTTCATTGCCTTTTATTGCAATGGATAGTTCTGGTCAACCAATTAATTTTGAAAAAGAATTAAATCGTGC
AACATTCGACAATTTAACTAAAAATTTAATTGAAAGATTAAAAAAACCTGTTTTAGATGCAATGAAAGAATCTA
AACTATCACTTGCTGATATTGACCAAGTTTTAATGGTTGGTGGTTCAACACGTATGCCAGCTGTACAAAATTTA
GTTAAA---
GAATTAACGGGCAAAGAACCAAATCATTCATTAAATCCTGATGAAGTTGTTGCGATCGGTGCAGCTATTCAAG
GTGGGGTATTAGCTGGAGAAATTGA

>Clostridium perfringens BA000016

ATTATATAGCAGAAGACTTTAAAGCTCAAAAC---GGAATTGATTTAAGACAAG--------------------------------------
----

ATAAAATGGCTCTTCAAAGATTAAAAGAAGCTGCTGAAAAAGCTAAAATTGAGTTATCATCATCAACTCAAAC
ATTAATCAACTTACCATTTATAACTGCTGATGCAACT---
GGTCCAAAACACATAGATATGACATTAACAAGAGCTAAATTCAATGAATTAACTCATGACTTAGTTGAAAGAA
CAATCAACATAATGAAAGAAGCCTTAAAATCAGGTAATGTTTCATTAAATGATATAGATAAAGTAATCTTAGTT
GGTGGATCAACAAGAATACCAGCAGTTCAAGAAGCTGTTAAA---
AACTTCACTGGAAAAGAACCTTCAAAAGGAGTTAACCCAGATGAGTGCGTAGCAATGGGTGCTGCTATCCAA
GCTGGTGTATTAACTGGTGATGTTAA
