# Supplementary material for: Non-ribosomal phylogenetic exploration of Mollicute species: New insights into haemoplasma taxonomy
Source: Infect Genet Evol. 2014 Apr;23(100):99–105. doi: 10.1016/j.meegid.2014.02.001 (PMC3988868; doi:10.1016/j.meegid.2014.02.001)
Supplement: Supplementary figure C — Fig. C. Concatenated sequence alignment. [file mmc3.pdf]

Supplementary Fig. C: Concatenated sequence alignment

GapA and *dnaK* concatenated sequence alignment. Sequences were aligned using MAFFT online multiple sequence alignment programme. Sequences are in fasta format.

>*Mycoplasma haemofelis* str. Langford1

ATTGGCTATTAGAGGAAATCAAGAAGGAACAC---TCTATAGATCTTTCTTCAG--------------------------------------
----
ATAATTTAGCACTTCAAAGACTTAAGGATGCCGCTGAGAAGGCAAAGATAGAGCTTTCTTCCGTTACTCAAAC
TCAAATACTCCTTCCGTTTCTTTCTATGGTGGGTGG---
ACAACCACTTAACATAGATAAAGTTGTTACTCGTGTTCAGTTTGAATCTTTAACTAAGCATTTAATTGAGAAGA
CTAGGAAGCCCTTCTTGGATGCTTTGAAGGAATCTAAGTTATCTGCTTCCGATATAGATCAAATTCTGTTGGTG
GGTGGTTCTACCCGTATGCCTGCCGTTCAGGAGTTGGTTAA---
GAGCCTTTCCGGGAAGACACCTAACTTGTCTATTAATCCCGATGAAGTTGTTGCTTTAGGTGCTTCCGTTCAAG
GTGCTATTCTTGCTGGGGATATTAACAACGACTTGACTGATCCTAAGACACTTGCTCACCTTCTTAAATATGAT
ACAGCTCATGGACCTGTTAGATGCTAT---GATATCAGTGTT------------GAAGGTGACAG---
TATTGTTTTAGTTAATAAATGTAGTGGAGAAAAACAATCCTTCAAAGTTATTTCTGAAAGAGATCCTAAAGCTC
TTCCTTGGAAGTCTTTAAATGTAGATTGCGTTCTTGAATGTACTGGTCGTTTTACCGATAAAGATGCAGCTATG
GCTCATGTTGAAGCGGGGGCTAAGAAAGTAGTTATCTCCGCTCCAGCAAAAGG---
TGATTTAAAGACAATCGTTTACAACGTAAACCATGGTACTTTAACTTCTTCTGATCAAGTTATCTCAGCAGCTTC
CTGTACAACTAACGCTTTAGCTCCCGTTGTAGATGCTCTTCACAAGAAGTACAAAATTGTTTCTGGGTTTATG

>*Mycoplasma haemofelis* str, Ohio2

ATTGGCTATTAGAGGAAATCAAGAAGGAACAC---
TCTATAGATCTTTCTTCAGCTCAAACTCAAATACTCCTTCCGTTCCTTTCTATGGTGGGTGGACAACCACTATAA
TTTAGCACTCCAAAGACTTAAGGATGCCGCTGAGAAGGCAAAGATAGAGCTTTCTTCTGTTA--------------------
----
TAACATAGATAAAGTTGTTACTCGTGTTCAGTTTGAATCTTTAACTAAGCATTTAATTGAGAAAACTAGAAAGC
CCTTCTTGGATGCTTTGAAGGAATCTAAGTTATCTGCTTCCGATATAGATCAAATTCTGTTGGTGGGTGGTTCT
ACCCGTATGCCTGCCGTTCAGGAGTTGGTTAA---
GAGTCTTTCTGGGAAGACACCTAACTTGTCTATTAATCCCGATGAAGTTGTTGCTTTAGGTGCTTCTGTTCAAG
GTGCTATTCTTGCTGGGGATATTAATAACGACTTGACTGATCCTAAGACACTTGCTCACCTTCTTAAATATGATA
CAGCTCATGGACCTGTTAGATGCTAT---GATATCAGTGTT------------GAAGGTGACAG---
TATTGTTTTAGTTAATAAATGTAGTGGAGAAAAACAATCCTTTAAAGTTATTTCTGAAAGAGATCCTAAAGCTC
TTCCATGAAAATCTTTAAATGTTGATTGTGTTCTTGAGTGTACTGGTCGTTTTACCGATAAAGATGCAGCTATG
GCTCACGTTGAAGCGGGGGCTAAGAAAGTGGTTATCTCTGCTCCAGCAAAAGG---
TGATTTAAAGACAATCGTTTACAACGTAAATCATGGCACTTTAACTTCTTCTGATCAAGTTATCTCAGCAGCTTC
CTGTACAACTAACGCTTTAGCTCCAGTTGTAGATGCTCTTCACAAGAAGTACAAAATTGTTTCTGGGTTTATG

>*Mycoplasma haemocanis* str. Illinois

ATTGGCTATTGGACGAGATTAAAAAGGAACAT---TCTATAGATTTATCTTCCG--------------------------------------
----
ACAGTTTGGCTCTTCAGAGATTGAAAGATGCCGCTGAGAAGGCAAAGATAGAACTTTCTTCTGTTACCCAAAC
ACAAATACTTCTTCCATTCCTTTCTATGGTAGGAGG---

GCAGCCATTGAATATAGATAAGGTAGTTACTCGTGTTCAGTTTGAATCTTTGACTAAACATTTGATTGAGAGAA
CTAGAAAACCATTTTTGGATGCTTTACAAGAGTCTAAATTGTCTGCTTCCGATATAGATCAGGTCTTGTTGGTG
GGTGGTTCTACCCGTATGCCTGCCGTGCAAGAGTTGGTTAA---
GAGCCTTTCTGGAAAAACTCCTAACTTGTCTATTAATCCGGATGAAGTGGTAGCTTTAGGTGCTTCTGTTCAAG
GTGCTATTCTAGCTGGAGATATTAAAAATGATTTAACTGATCCGAAGACACTTGCTCACCTTCTTAAATATGAT
ACAGCTCATGGTCCTGTTAAGTGTTAC---GATATCAGTGTT------------GAAGGTGACAG---
TATTGTTTTAGTTAATAAGTGTAGCGGGGAAAAGCAGTCTTTTAAAGTTATTTCCGAAAGAGATCCTAAAGCTT
TACCTTGAAAATCTTTGGGTGTAGATTGTGTTCTTGAATGTACTGGACGCTTTACTGATAAGGATGCAGCCATG
GCTCACGTTGAGGCTGGAGCTAAGAAAGTGGTTATCTCTGCTCCAGCAAAAGG---
AGATCTTAAGACAATCGTTTACAACGTAAACCACAATACTTTAACTTCCTCTGATCAAGTTATTTCCGCTGCTTC
TTGTACCACTAATGCTTTAGCTCCCGTTGTAGATGCTCTTCATAAGAAGTACAAAATTGTTTCTGGTTTTATG

>*Mycoplasma haemomuris*

ACTGGATCGTTAAAGARGTTCAAAGAGAACAC---GGTGTTGACCTTTACTCKG-------------------------------------
----
ACAACCTGGCTCTTCAAAGGGTTAAGGACGCTGCTGAGAAGGCTAAGATYGAACTTTCCTCCGTGACCCAATC
CCAAATCCTTCTTCCGTTCATCTCCATGGCGGGTGG---
ATCTCCTCTTAACGTTGAGAAAACACTTACAAGGGTTGAGTTCGAATCCCTAACCAAACACCTTCTTGAGAAGA
CAAGACAACCTTTCCTTGACGCYCTKAAAGARGCTAAACTTGAGTCCAAGGACGTTGACCAAATCCTTCTKGTT
GGTGGTTCCACCAAGATGCCATGTGTCCAAAAACTTGTTAG---
GGATCTGTCCGGCAAAAACCCAAACCTATCCATCAACCCAGACGAGGTTGTTGCCCTAGGTGCTTCGGTTCAA
GGTGCTATCCTTGCGGGTGACATCAACAATGACCTAACCGATGCCAAAACCCTTGTTCACCTTCTTAAATATGA
CTCGGCCCACGGACCACTTTGCTGCTGCTGGAAGGTTGAGGTT------------
CAAGAGAACAAGTTCATTCTTAGAAGCAGCGAAAAAACCGT---------
TGAAGTTAAGGTATTTGCAGAAAGAGATCCCGCTAACCTACCTTGGGGAGATCTTGGAATCGACTGCGTAATC
GAAAGTACAGGTAGATTCACCGACAAAGACGCAGCTTCCGCCCACATTAAAGCCGGTGCAAGAAAGGTTGTT
ATCTCGGCTCCGGCTAAAGG---
TGACCTTAAAACCATCGTCTTCAACGTTAACCACAATGTTCTTACAAAAGACGACCAAGTTATCTCCGCAGCTT
CCTGTACAACCAACGCTCTTGCCCCAATCGTAAGCGCACTCCACAATTCCTTCGGAGTGGTTTCGGGCTTCATG

>*Mycoplasma suis* str. Illinois

AGTGACTTTTGGAATCAATCCAAAAGGAACAT---AGTGTAGACCTATCTAAAG------------------------------------
----
ATAACTTAGTAATGCAAAGACTAAAGGAAGCTGCTGAAAAAGCAAAGATTGAACTTTCTTCAGTTCAACAAAC
ACAAATTATGCTTCCTTTCCTTTCAATGGTTCGTGG---
AGAACCATTGAATGTTGATTTCTCATTAACTAGAGAACAATTCCAACTATTTACTAAGGATTTACTAGAAAGAA
CAATAGCACCTGTTAAGGATGCTATTGCAGAATCTAAGTTATCACTTTCAGACATAAATGAAGTTCTACTAGTA
GGTGGTTCTACTAGAATGCCTGCAGTACAAGAACTAGTAGA---
AAAACTAACTGGAAAGAAACCTAATTTGTCTATTAATCCAGATGAAGTAGTAGCTCTAGGAGCTTCTGTTCAA
GCTGGAATTCTAGCAGGAGATATCAATAATGACGTAGTTGACATTAAAGTTCTTACTCATCTTTTGGTTTATGA
CAGTGCTCAAGGAAAACTAAAAGATTGA---GAAGTAAGTTGT------------GATTCAGAATA---
CATAAGACTAAAGAATGTAAACACCGGAGAAGTTAGAGAAGTTAGAGTTTTCAACTTCAATACTGAAAAGATT
TATCACTGAGGTGAACTAGAAATTGATTGTGTTGTTGAATGTTCAGGAAGATTCTTGACTAAGGAAGCGGTTA
AGTGTCACCTTGATGCAGGAGCTCAAAAAGTTCTTATTTCAGCCCCAGCAAAGGA---

TGACACTAAGACAGTTGTTTACAACGTAAACCACACTCAAATTACTAGTTCAGACAATGTTATTTCAGGAGCTT
CATGTACAACTAATGCATTAGCTCCTATCGTAAAAATTATTCACAGAAAATTTGGAATTAATTCTGGATTCATG

>*Mycoplasma suis* str. KI_3806

AGTGACTTTTGGAATCAATCCAAAAGGAACAT---AGTGTAGATCTATCTAAAG-------------------------------------
----

ATAACTTAGTAATGCAAAGATTAAAAGAAGCTGCTGAAAAGGCAAAGATTGAACTTTCTTCAGTTCAACAAAC
ACAAATTATGCTTCCTTTCCTTTCAATGGTTCGTGG---
AGAACCATTGAATGTTGATTTCTCATTAACTAGAGAACAATTCCAACTATTTACTAAGGACTTACTAGAAAGAA
CAATAGCACCTGTTAAGGATGCTATTGCAGAATCTAAATTATCACTTTCAGATATAAATGAAGTTCTACTAGTA
GGTGGTTCTACTAGAATGCCTGCAGTACAAGAACTAGTAGA---
AAAATTAACTGGAAAGAAACCTAACTTGTCTATTAACCCAGATGAAGTAGTAGCTCTAGGAGCTTCTGTTCAA
GCTGGAATTCTAGCAGGAGATATTAATAATGACGTAGTTGACATTAAAGTTCTTACTCACCTTTTGGTTTATGA
CAGTGCTCAAGGAAAACTAAAAGATTGA---GAAGTAAGTTGT------------GATTCAGAATA---
CATAAGACTAAAGAATGTAAATACTGGAGAAGTTAGAGAAGTTAGAGTTTTCAACTTCAATACTGAAAAGATT
TATCACTGAGGTGAATTAGAAATTGATTGTGTTGTTGAATGTTCAGGAAGATTCTTAACTAAGGAAGCAGTTA
AGTGTCACCTTGATGCAGGAGCTCAAAAAGTTCTTATTTCAGCTCCTGCAAAGGA---
TGACACTAAGACAGTTGTTTACAACGTAAACCATACTCAAATTACCAGCTCAGACAATGTTATTTCAGGAGCTT
CATGTACAACTAATGCACTAGCTCCTATCGTAAAAATTATTCACAGAAAATTTGGAATTAATTCTGGATTCATG

>*Candidatus* Mycoplasma haemominutum str. Birmingham1

ACTGATTACTTGAAACTATTAAAAAAGAACAT---GGAGCTGATCTCTCAAAGG-------------------------------------
----

ATAATCTTGTGTTACAAAGATTAAAAGAAGCAGCAGAAAAAGCAAAAATAGAACTTTCTTCAGTGCAACAAAC
TCAAATTATGTTGCCCTTCCTAACTATGATTGGAGG---
AGAGCCTCTAAATGTAGACTTAACTCTTTCTAGAGCTCAATTTGAATTACTAACAAAAGATTTACTAGATAGAA
CAGTAAGACCTGTAGAAGATGCTGTCAAAGAATCTCAACTGAAATTAAGTGATATAGATCAAATACTTCTAGT
AGGAGGCTCTACTAGGATGCCAGCAGTGCAAGCACTTGTAGA---
AAAATTAACTGGAAAGAAACCTAATCTTTCAATAAATCCTGATGAGGTAGTAGCATTGGGAGCAGCTGTTCAA
GCAGGAGTACTAGCAGGAGATGTAAATAATGATATTGTTGATGCATCGGTTTTAGCTCATTTATTAAAGTATG
ACAGCTCTCAAGGAGTGTTGAAAGATTGA---GAGGTTAAAAGT------------GATGCAGAAAA---
CATTTACTTAACTCACATTGATAGCGGAAAACAAAAAACAGTGAAGGTCTTTAATTTCTTGAAAGAAAAGAGC
TACCACTGGGGAGAGTTAGAAGTTGATTGCGTGGTGGAATGTTCGGGACGTTTATTAACTAAAGATGCAGTG
CAATGCCACTTGGATGCAGGAGCTGAGAAAGTATTAATCTCAGCTCCTGCAAAAGATGATGCTATAAAAACTA
TTGTTTTTAACGTAAATCATAATTCGATTAGCACTTCTGATACGGTAATTTCTGGAGCTTCTTGCACTACCAATG
CATTGGCTCCTGTTGTTAAAGTGTTGCATAGAAAATTTGGAGTGCAGTCTGGATTTATG

>*Candidatus* Mycoplasma erythrocervae

CTTGATTGTTAGATAGCATAAAGAAAGAACAT---GATGTGGATCTATCTAAAG------------------------------------
----

ATAATTTGGTAATGCAAAGATTGAAGGAATCTGCTGAAAAAGCCAAGATTGAACTTTCCTCAGTTCAACAAAC
TCAAATTATGTTACCTTTCTTAACAATGGTTAAAGG---
AGAGCCCTTGAATGTTGATTTAACTCTTTCCAGATCTCAATTTGAGCTTTTAACAAAAGATCTGTTAGATAGAA
CTGAAAAGCCAGTTTTGGATGCTATTGCAGAGTCAAAAGTTGAGTTAAACAAGATCGATGAAGTTCTTTTGGT

AGGAGGTTCTACTAGAATGCCAGCTGTTCAACAATTAGTAGA---
AAGATTAACTAATAAGAAACCTAATCTTTCTATTAATCCTGATGAAGTGGTAGCTTTGGGAGCTTCTGTTCAAG
CTGGTATTTTGGCTGGAGATATTAAAAATGACATTGTTGACACAACAGTATTAGCTCACCTTCTGAAATATGAC
AGTTCACAAGGTGTTCTAAAGGATTGA---GAAGTTACTTGT------------GATCAAGAAAA---
GATTTATTTGAAAAACTTAAATAGCGGAGCAGAAAAAAGCTTCAAAGTGTACAACTTTACCAAAGAAAAGACT
TATCACTGAGGAGAACTAGATGTTGATTGTGTAGTTGAATGTTCAGGGAGACTATTAACTAGAGATGCGGTTC
AATGTCATTTAGACGCTGGAGCAGATAAGGTATTAATTTCAGCTCCAGCTAAGGATGACAGTATTAAGACTGT
TGTTTTCAATGTAAATCACAACTCTATTGCCACTTCAGATAATGTAATTTCTGGAGCTTCTTGTACTACAAATGC
GCTTGCTCCTGTAGTTAAGGTTCTTCACAGAAAATTTGGAATCCAATCTGGCTTTATG

>*Mycoplasma wenyonii*

ATTGATTACTTGAAACTATCAAGAAGGAAAAC---AATGTAGATCTTTCAAAAG--------------------------------------
----
ATAACTTAGTAATGCAAAGATTGAAAGAGTCTGCAGAAAAGGCAAAGATTGAACTTTCTTCAGTTCAACAAAC
TCAAATTATGTTGCCTTTCTTAACAATGATTAATGG---
AGAGCCTCTAAATGTTGACTTAACTCTCTCTAGATCACAATTTGAGTTATTAACTAAAGATTTACTAGATAGAA
CTGAAAAGCCTGTTCTGGATGCAATTAGAGAATCAAAGATTGAGTTAAACCAAATTGATGAAGTTCTTTTGGT
TGGGGGTTCAACCAGAATGCCAGCAGTTCAAGGATTAGTTGA---
AAGATTAACTAAGAAGAAACCAAACCTATCTATTAATCCTGATGAGGTTGTAGCCCTAGGAGCTTCAGTTCAA
GCTGGTATCTTAGCTGGAGATATTAATAATGATATTGTTGAACCAAGTGTATTGGCACACTTACTAAAGTATGA
CAGCTCACAAGGTGTATTGGTTGATTGG---GAAATTAGTAGT------------GATCAAGAAAA---
TATTCACTTAAAGAATCTAAAGAGTGGAGTAGAAAAGAGTTTCAAGGTTTACAACTTCAATAAGGAAAAGAGT
TACCACTGAGGAGAATTAAATGTTGACTGTGTAGTTGAATGTTCTGGTAGGTTACTAACTAGAGAGGCAGTTA
AGTGTCACTTGGATGCTGGTGCAGATAAGGTATTAATTTCTGCTCCTGCAAAGGATGACAGCATTAAGACTGT
TGTATACAATGTGAACCACAACTCAATTGCAACTTCAGATAATGTAATTTCTGGAGCTTCCTGTACTACAAATG
CTTTAGCTCCTGTAGTAAAGGTATTACACAGAAAGTTTGGAATTCAATCAGGATTTATG

>*Candidatus* Mycoplasma haemocervae

GTTGATTAATTGACACTATCAAAAAAGAACAT---GGTGTAGACGTATCTAAAG------------------------------------
----
ATAATTTGGTATTGCAAAGATTGAAAGAAGCTTCAGAAAAAGCAAAGATTGAATTATCCTCAGTTCAACAAAC
TCAAATCATGTTGCCTTTCCTAACAATGGTTAATGG---
AGAACCTTTGAATGTTGACTTAACTCTTTCAAGATCTCAATTTGAACTTCTTACAAAAGATCTATTAGAAAGAAC
TGAAAAGCCTGTTTTGGACGCAATTAAGGAATCAAAGATTGAACTAAATCAAATAGATGAAATTCTTTTGGTG
GGTGGATCTACTAGAATGCCTGCTGTTCAAGAATTGGTTGA---
AAAGTTAACTAAAAAGAAGCCTAATCTATCTATAAACCCAGATGAAGTAGTCGCTCTAGGTGCTTCTGTTCAA
GCTGGAATTTTGGCTGGTGACATAAATAATGACATTGTTGATCCTAGTGTATTAGCTCACCTACTAAAGTACGA
CAGCTCACAAGGAGTATTAACAGACTGA---GAAATCACTAGT------------GATCAAGAAAC---
TATTTACCTTAAGAATCTAAAGAGCGGAACACAAAAGAGTTTTAAGGTTTACAACTTTAACAAGGAAAAAAGT
TATCACTGAGGAGAACTTGATGTTGATTGTGTAGTTGAATGTTCAGGTAGATTATTAACTAGAGAAGCAGTTA
GATGTCACTTGGATGCTGGAGCAGACAAGGTATTAATTTCAGCTCCCGCTAAGGATGACAGCATCAAGACTGT
TGTTTACAACGTTAACCACAACTCAATTGCTACTTCAGATGATGTAATATCAGGAGCTTCTTGCACAACAAATG
CTCTAGCTCCTGTTGTTAAGGTACTTCACAGAAAGTTTGGAATCCAATCTGGTTTCATG

>*Mycoplasma ovis*

ATTGATTAATTGAAACTATTAGAAAGGAACAT---GGCGTAGATCTTTCTAAAG-------------------------------------
----
ATAATTTGGTAATGCAAAGGTTGAAGGAAGCTTCAGAAAAGGCAAAGATTGAATTATCTTCAGTTCAACAAAC
TCAAATAATGTTGCCCTTCTTAACAATGGTTAACGG---
AGAACCTTTAAATGTTGATTTAACTCTTTCAAGATCTCAATTTGAGCTTCTTACAAAAGACTTATTAGATAGAAC
TGAAAGACCTGTTCTAGATGCAATTAAAGAATCGAAGATTGAATTAAAGCAAATAGATGAAGTTCTTTTGGTT
GGCGGCTCTACTAGAATGCCAGCTGTTCAAGCATTAGTTGA---
AAAGTTAACTGGAAAAAAACCTAATTTATCTATAAATCCGGATGAAGTAGTCGCTTTGGGCGCTTCTGTTCAG
GCTGGAATTTTGGCTGGTGATATAAATAATGACATCGTTGAACCTAGTGTATTGGCACACTTGTTAAAGTATG
ACAGCTCACAAGGAGTATTAACAGATTGG---GAAGTAACTAGT------------GATCAAGAAAC---
TATTTATCTTAAGAATTTAAAAGGAGGGGGAACAAAAAGTTTTAAAGTTTACAACTTTAACAAAGAAAAGAGT
TATCACTGAGGAGAGCTGGATGTTGATTGTGTAGTTGAATGTTCAGGTAGATTATTAACCAGAGAAGCAGTG
AGATGTCACTTGGATGCTGGTGCAGACAAGGTATTAATTTCAGCTCCAGCTAAGGATGACAGCATCAAGACTG
TGGTTTTCAATGTTAACCACAATTCCATTGCTACTTCGGATGATGTAATCTCAGGAGCTTCTTGTACAACAAATG
CTTTAGCTCCTGTTGTTAAGGTACTTCACAGAAAGTTTGGAATCCAATCTGGCTTCATG

>*Candidatus* Mycoplasma haemolamae

ACTGATTAAAGGAAACCATTCACAAAGAACAT---GGCGTTGATGTTTCTAAAG-------------------------------------
----
ACAATCTTGTTATGCAACGTCTAAAAGAAGCTGCTGAGAAGGCAAAGATAGAACTTTCGTCTGTACAACAAAC
TCAAATAATGTTGCCTTTCTTGACGATGATTCAGGG---
AGAACCTTTGAACGTTGATCTCTCTATGACTAGAGATCAATTCCAGATGCTCTCAAAAGATTTGCTAGATAGAA
CTGAAAAGCCTGTAAAGGATGCTATGAAAGAATCAGGCTTTGAACTTAAGGATATTGATGAAGTTTTGTTGGT
TGGAGGATCCACCAGAATGCCTGCTGTTCAAGATCTTGTAGA---
GAAGTTGACTAACAAGAAACCTAACCTATCTATCAACCCTGATGAAGTGGTAGCTTTGGGCGCTTCTGTTCAA
GCAGGTATCTTGGCTGGCGAAATTAAAAATGACATTGTTGACTCGAAAGTTCTTGCTCACCTTCTAAAGTACGA
CAGCTCTCAAGGGGTTCTTAGAGATTGA---GATGTTACCCAC------------GATCAAGATCA---
CATAAGACTAAAGCATATTTCAACAGGAGTTGAAAAGACAGTTAGAGTATTTGACTTTAGTAAGGAAAAGAG
CTATCACTGAGGAGAGTTAGAAATCGACTGTGTCGTTGAATGTTCAGGAAGACTATTAACTAGAGATGCTGTT
CAATGTCACCTTGATGCTGGAGCTGAAAAGGTTTTGATCTCGGCGCCTTCGAAGGATGAAAGCGTTAAGACAA
TTGTGTTTAACGTAAACCACAATTCGATTGCCACTTCAGATAACGTTATCTCGGGAGCATCTTGTACGACAAAC
GCTCTTGCTCCTATTGTTAAGGTACTACACAGAAAGTTTGGAGTTCAATCAGGATTTATG

>*Mycoplasma agalactiae* str. 5632

AATGATTAATTGATTTAATCAAAAAAGACTAT---AAAACAGATGTTACAAACA-------------------------------------
----
ATAAAATAGCAATGGCTCGTTTAAAAGCAGCAGCTGAAAAAGCAAAAATTGACTTATCTAGTTCACAACAAGC
TACAATTATGCTTCCATTTTTAGTTATGCAACAAGGCTCAGAACCTATTAGTGTTGAAGCAACCTTAAGAAGAA
GTCAATTTGAAGAAATGACATCACATTTGGTTGAAAGATGTAGAAAACCTATTGAAACAGCTTTAGCTGATGC
TAAAATCAAGATTTCTGATTTAGATGACGTAATTTTAGTTGGTGGCTCAACAAGAATTCCTGCAGTGCAACAAT
TAGTTGA---
ATCGATTTTAAATAGAAAAGCTAACCGTTCAGTTAATCCTGATGAAGTTGTAGCTATGGGTGCCGCTATTCAAG
GTGCTGTTTTAGCTGGCGAAATCGATAATGACTTAACTGAACCAAAAATGCTTGCACACTTATTAAAATATGAC
ACTGCTTTTGGCCCATT---AAAGGCT---GATGTAGTTGTT------------AAGGATGATGC---

TTTTATTGTAAATGGCAAAGAAATCAA------------------
GGTATTTTCTGAAAAAGATCCTGCCTTGCTGCCTTGAAAAGAACTTAATATTGATATTGTTTTAGAATGTACAG
GTTTTTTTGTTAAAAAAGATCTTGCTCACAAACACATAGAGGCTGGTGCTAAGAAAGTTATTATTTCAGCTCCT
GCTGGTAA---
AGACGTAAAAACTGTTGTTTATGGTGTAAACCATGAAATTCTATCTGCTGATGATGAAATTATCTCAGGGGCTT
CATGTACAACAAACTGTTTAGCTCCTGTAGTTAAGGTTTTAGTAGATAACTTTGGTATTGAAAATGGATTCATG

>*Mycoplasma agalactiae* str. PG2

AATGATTAATTGATTTAATCAAAAAAGACTAT---AAAACAGATGTTACCAACA---------------------------------------
----

ATAAAATGGCAATGGCTCGTTTAAAAGCGGCAGCTGAAAAAGCAAAAATTGACTTATCTAGTTCACAACAAGC
TACAATTATGCTTCCATTTTTAGTTATGCAACAAGGTTCAGAACCTATTAGTGTTGAAGCAACCTTAAGAAGAA
GTCAATTTGAAGAAATGACATCGCATTTAGTTGAAAGATGTAGAAAACCTATTGAAACAGCTTTAGCTGATGC
TAAAATCAAGATTTCTGATTTAGATGACGTAATTTTAGTTGGGGGTTCAACAAGAATTCCTGCAGTGCAACAAT
TAGTTGA---
ATCAATTTTAAATAAAAAAGCCAACCGTTCAGTTAATCCTGATGAAGTTGTAGCAATGGGTGCCGCTATTCAA
GGTGCTGTTTTAGCTGGCGAAATCGATAATGACTTAACTGAACCAAAAATGCTTGCGCACTTATTAAAATATG
ACACTGCTTTTGGCCCATT---AAAGGCT---GATGTAGTTGTT------------AAGGATGATGC---
TTTTATTGTAAATGGCAAAGAAATCAA------------------
GGTATTTTCTGAAAAAGATCCTGCCTTACTGCCTTGAAAAGAACTTAATATTGATATTGTTTTAGAATGTACAG
GTTTTTTTGTTAAAAAAGATCTTGCTCACAAACACATAGATGCTGGTGCTAAGAAAGTTATTATTTCAGCTCCT
GCTGGTAA---
AGATGTAAAAACTGTTGTTTATGGTGTAAACCATGAAATTCTATCTGCTGATGATGAAATTATCTCAGGTGCTT
CATGTACAACAAACTGTTTAGCTCCTGTAGTTAAGGTTTTAGTAGATAACTTTGGTATTGAAAATGGATTCATG

>*Mycoplasma arthritidis*

AATGAATGGTTGAAGAAATTAAAAATAAATAC---AACTATAACCCAGAAACTG--------------------------------------
----

AAAAAATGGCAATGGCAAGACTAAAAGAAGAAGCTGAAAGAGCAAAAATTACTCTTTCTGAAAGTATGGTTG
CTAATATTTCTCTACCATTCTTGGCCATGTCACAAAC---
AGGACCAATTAATGTCGAATTGGAACTAAAAAGATCGGAGTTTGAAAAAATGACTGACGATCTACTACAAAG
AACTAAAAAACCACTATTAGATGCATTAAGCGAAGCAAAATTACAAATTAATGATCTAGATGAAGTTTTATTAG
TTGGTGGTTCAACTAGAATGCCCGCTGTTCAAAAATTAGTAGC---
TGATACCATTAATAAAAAACCTAACAATTCTATTAACCCTGATGAAGTAGTTAGTGTTGGAGCCGCTATTCAAG
GAGCTATCTTAGCTGGTGATGTTCATAATGATTTAACAGATGCCAAAACTTTAGCACATTTACTAAAATACGAC
ACAGCACACGGAAAAAT---GAATGCA---GAAATCTCACAC------------ACCGAAAATTC---
AATCATTGTTGACGGTGTGGAATATCC------------------
TGTTTATGCTGAAAAAGATCCTGCAATGCTACCTTGAAAAAAACTTGGTGTTGATATTGTAGTTGAAGGAACT
GGTCGTTTCGTTACCGTTGAAGGTTCACAAAAACACATTGAAGCAGGAGCTAAAAAAGTTTTAATTACCGCTC
CTTCAAAAACTGATAGCGTTAAAACAATAGTTTATTCAGTAAATGAAAATACTTTAAATAAAGACGATGTTATT
GTTTCAGCAGCATCATGTACAACTAACTCTTTAGCACCAGTTATGAATGTCTTAGAAAATGAATTTGGTGTAGT
TAAAGGATATATG

>*Mycoplasma hominis*

AATGAATGGTTGAACAAATTAAATCTAAATAC---AATTTCGATCCAACAACTG-------------------------------------
----

ACAAAATGGCAATGGCAAGATTAAAAGAAGAAGCAGAACGTGCAAAAATTACTTTATCAGAACAATTAATTG
CAAACATTTCTCTTCCATTCTTAGCAATGAATGAAAA---
TGGACCAGTTAATGTTGAATTAGAAATTACTCGTGCTACATTTGAATCAATGACTGAACATTTACTACAAAGAA
CAAGAAAACCATTATTAGATGTTTTAAGTGAAGCAAAATTAACATGAAATGATATTAATGAAGTATTACTAGTT
GGTGGTTCAACAAGAATGCCAGCTGTTCAAAAACTAGTAGC---
AGAAGTAACTAATAAAAAACCAAATAATTCAATTAACCCAGATGAAGTAGTTAGCGTTGGAGCTGCTATTCAA
GGTGCAATATTAGCAGGAGAAATTCATAATGATTTAACAGATGCAGCTACATTAGCTCACTTATTGAAATACG
ATACAGCTCATGGAAAAAT---GAATGAA---AATATTTGACAC------------ACAGAAAATTC---
TATTGTTGTAAATGGCAAAGAATATCG------------------
TGTTTATAGTGAAAAAGATCCATTAAACCTACCTTGAAAAGAACTAAACGTAGATATAGTTATTGAAGGAACT
GGAAGATATGTAACAAAAGAAGGTGCTGAATTACATATTCAAGCAGGTGCTAAAAAGGTGTTTATTACTGCTC
CAGCTAAAAGCGAAGGTGTTAAAACAGTTGTTTATTCAGTAAACGAAGATATCATTACGCCAGAAGATAAAAT
TTTATCAGGCGCTTCATGTACTACTAACTGTTTAGCTCCTATTGCCAACGTATTGGAAAAAAACTTTGGTATTGA
AAAAGGATTTATG

>*Mycoplasma conjunctivae*

ATTGAATGGTAGCTAAAATTAAACAAGAATAC---AACTTTGATGCAACTAAAG-------------------------------------
----

ACAAAATGGCTTTATCTAGATTAAAAGAAGAAGCTGAAAAAGCAAAGATTAACTTATCAAACCAAATGGTTAC
CACAATTACACTACCTTTTTTAGGTATGAATGAAAA---
TGGACCAATTAATGTTGAACTTGAGCTCAAAAGATCTGAATTTGAAAAGCTTAGCGCCCACTTGGTAGACCGT
ACTAGAAAACCAATTGTTGATGCGCTCAAAGAAGCCAAATTAGAAGCTAAAGACCTCGATGAAATTTTATTAG
TTGGAGGTTCAACTCGAATTCCTGCTGTCCAAACAATGATTGA---
GCACACCTTAAATAAAAAGCCAAATCGCTCAATTAATCCAGATGAGGTAGTAGCTATTGGAGCTGCTATTCAA
GGGGGGGTTTTAGCTGGTGAAATCAACAATGATTTAACAGATGTTAAAACCTTAGCACACCTATTTAAATATG
ATACAGCTCATGGAAAATT---TGAAGGT---AAAGTTGATTATATTCAAGAAGAAGACAAAAGCTT---
TTTAGTAATTAACGGACACAAAATTTT------------------
AATTCTTTCCCAAAGAGATCCAAAAACACTTCCTTGAGCTCAATTAGGAATCGATATTGTGCTTGAATGTACAG
GATTTTTCGCTTCCAAAACAGGAGCACAATTACACCTAGACGCTGGTGCTAAAAAAGTTGTTATTTCTGCACCT
GCAGGAAA---
CGATGTTAAAACTATTGTCTACAATATTAACCACGAAACAATTACCGAAGAGGATACAATCTTATCAGCGGCAT
CATGTACCACAAACGCTTTAGCACCGGTTGTAAATGCACTTGAAAAAGAATTTGGAATTGAAAATGGATATAT
G

>*Mycoplasma hyopneumoniae* str. 232

ATTGACTTGTTAAAAAAATCAAAGAAGTATAT---GATTTTGATCCAAAAAGTG-------------------------------------
----

ATAAAATGGCGCTTACAAGACTTAAAGAAGAGGCTGAAAAAACCAAAATTAATCTTTCAAATCAAAGTGTTTC
TACAGTTTCTCTACCATTTTTAGGAATGGGCAAAAA---
CGGGCCGATTAACGTTGAACTTGAACTTAAAAGATCAGAATTTGAAAAAATGACTGCCCATTTAATCGATAGA
ACTCGCAAACCAATTGTTGATGCTCTAAAACAAGCAAAAATTGAGGCTTCAGATCTTGATGAAGTTCTCCTTGT
AGGTGGATCAACAAGAATGCCAGCTGTTCAGTCAATGATTGA---

GCATACTTTAAATAAAAAGCCAAATCGTTCAATTAATCCTGATGAGGTAGTCGCAATTGGTGCTGCAATTCAA
GGGGGGGTTCTAGCTGGAGAGATCAGTAACGACTTAACAGATGCATCAGTTTTAGCTCATTTATTTAAGTATG
ATTCCGCCCATGGAAAGTT---TAACGGT---GAAGTTGAAGTTTTAAAAGATAACGGAAAAAATTA---
CCTTAAAATTAAAGGTCAAAAAATTCT------------------
TGTTTTATCTGAAAGAGACCCAAAGTCCTTGCCTTGAGGTCAGCTTGGAATTGATTTGGTTGTTGAATGTACAG
GATTTTTTGCTTCAAAATCAGGAGCTAGTCAACATTTAGAGGCGGGAGCAAAAAAAGTAATAATTTCTGCTCC
GGCAGGAAA---
TGATGTTAAAACCATTGTTTATAACGTAAATTGTGATACAATTACTGAAGATGATAGAATTTTATCCTCAGCTTC
TTGCACTACAAACGCACTTGCCCCACTTGTAAATGCGCTTGATAAAGAATTTGGGATAAACCACGGATTTATG

>*Mycoplasma hyopneumoniae* str. 7448

ATTGACTTGTTAAAAAAATCAAAGAAGAATAT---GATTTTGATCCAAAAAGTG---------------------------------------
----
ATAAAATGGCGCTTACAAGACTTAAAGAAGAGGCTGAAAAAACCAAAATTAATCTTTCAAATCAAAGTGTTTC
TACAGTTTCTCTACCATTTTTAGGAATGGGCAAAAA---
CGGGCCGATTAACGTTGAACTTGAACTTAAAAGATCAGAATTTGAAAAAATGACTGCCCATTTAATCGATAGA
ACTCGCAAACCAATTGTTGATGCTCTAAAACAAGCAAAAATTGAGGCTTCAGATCTTGATGAAGTTCTCCTTGT
AGGTGGATCAACAAGAATGCCAGCTGTTCAGTCAATGATTGA---
GCATACTTTAAATAAAAAGCCAAATCGTTCAATTAATCCTGATGAAGTAGTCGCAATTGGTGCTGCAATTCAAG
GGGGGGTTCTAGCTGGAGAGATCAGTAACGACTTAACAGATGCATCAGTTTTAGCTCATTTATTTAAGTATGA
TTCCGCCCATGGAAAGTT---TAACGGT---GAAGTTGAAGTTTTAAAAGATAACGGGAAAAATTA---
CCTTAAAATTAAAGGTCAAAAAATTCT------------------
TGTTTTATCTGAAAGAGACCCAAAATCCTTGCCTTGAGGTCAACTTGGAATTGATTTGGTTGTTGAATGTACAG
GATTTTTTGCTTCAAAATCAGGAGCTAGTCAACATTTAGAGGCGGGTGCAAAAAAAGTAATAATTTCTGCTCC
GGCAGGAAA---
TGATGTTAAAACCATTGTTTATAACGTAAATTGTGATACAATTACTGAAGATGATAGAATTTTATCCTCAGCTTC
TTGCACTACAAACGCACTTGCCCCACTTGTAAATGCGCTTGATAAAGAATTTGGGATAAACCACGGATTTATG

>*Mycoplasma hyopneumoniae* str. J

ATTGACTTGTTAAAAAAATCAAAGAAGAATAT---GATTTTGATCCAAAAAGTG---------------------------------------
----
ATAAAATGGCGCTTACAAGACTTAAAGAAGAGGCTGAAAAAACCAAAATTAATCTTTCAAATCAAAGTGTTTC
TACAGTTTCTCTACCATTTTTAGGAATGGGCAAAAA---
CGGGCCGATTAACGTTGAACTTGAACTTAAAAGATCAGAATTTGAAAAAATGACTGCCCATTTAATCGATAGA
ACTCGCAAACCAATTGTTGATGCTCTAAAACAAGCAAAAATTGAGGCTTCAGATCTTGATGAAGTTCTCCTTGT
AGGTGGATCAACAAGAATGCCAGCTGTTCAGTCAATGATTGA---
GCATACTTTAAATAAAAAGCCAAATCGTTCAATTAATCCTGATGAGGTAGTCGCAATTGGTGCTGCAATTCAA
GGGGGGGTTCTAGCTGGAGAGATCAGTAACGACTTAACAGATGCATCAGTTTTAGCTCATTTATTTAAGTATG
ATTCCGCCCATGGAAAGTT---TAACGGT---GAAGTTGAAGTTTTAAAAGATAACGGAAAAAATTA---
CCTTAAAATTAAAGGTCAAAAAATTCT------------------
TGTTTTATCTGAAAGAGACCCAAAGTCCTTGCCTTGAGGTCAGCTTGGAATTGATTTGGTTGTTGAATGTACAG
GATTTTTTGCTTCAAAATCAGGAGCTAGTCAACATTTAGAGGCGGGAGCAAAAAAAGTAATAATTTCTGCTCC
GGCAGGAAA---

TGATGTTAAAACCATTGTTTATAACGTAAATTGTGATACAATTACTGAAGATGATAGAATTTTATCCTCAGCTTC
TTGCACTACAAACGCACTTGCCCCACTTGTAAATGCGCTTGATAAAGAATTTGGGATAGACCACGGATTTATG

>*Mycoplasma pulmonis*

AATGATTAACTAAAGAAATTAAAAATAGATAT---GATTTTGACCCTTCAAAAG-------------------------------------
----

ACAAAATGGTAATGACAAGACTTAAAGAAGCAGCTGAAAAAGCAAAAATTGACCTTTCAGCACAAATGGTAG
CTCAAATTACTCTTCCTTTCTTGTCAGTTACTTCTAA---
AGGACCTATTAACGTTGATTTAGAACTAAAAAGAAGTGAATTTGAAAAAATGACAACTCATTTAGTTGATAGA
ACTAGAAAACCTATTGAAGATGCTCTAAGAGAGGCAAAAATTAAAGCAAGTGACCTTAGTGAAGTTCTTTTAG
TTGGAGGATCAACTAGAATTCCTGCTGTTCAATCTATGGTAGA---
GCATGTTCTTGGTAAAAAACCAAATCGTTCAATTAACCCTGATGAAGTTGTTGCTATTGGAGCTGCAATTCAAG
GTGGAGTTTTAGCAGGAGATATCAAAAATGACTTAACTCAATCTTCAACTTTAATGCACTTATTAAAATTTGAC
ACAGCATATGGAAGATT---TAACTCA---AAAGTTGAATTA------------ACAGAAAAAGG---
ATTTTCAGTAGATTCAAAAGAAGTTCT-----------------
AGTTTTTGCAGAAAGAGACCCAAGAAACTTACCTTGAGGAAAATTGGAAATTGACCTTGTTCTAGAGTGTACA
GGAATGTTTGCTTCAAAAGAAAAATCTCAAGTTCACCTTGATGCAGGGGCTAAAAGAGTTCTTATTTCAGCTCC
AAGTGGCTC---
AGATGTTAAAACTATTGTTTATGGAGTTAATGACTCTTCTCTTTCTTCAGAGGATAAAATTGTCTCAGCTGCTTC
ATGTACAACAAACTGCTTGGCTCCACTTGTAAATGCGCTTGAAAAAGAATATGGAATTCTAACAGGTTCTATG

>*Mycoplasma crocodyli*

AATGATTAACAGGTCTTATTAAAAAAGAACAC---AATTTTGATGCATCAACCG-------------------------------------
----

ATAAAATGGCTATGGCTCGTTTAAAAGAAGCTGCAGAAAAAGCAAAAATCGATTTATCAAACCAATCAATTGC
TACAATTAACTTACCTTTCTTAGCGGTTACACCATC---
AGGACCTATTAACGTTGAAGTTGAATTAAAACGTAGTGAATTCGATGCTATGACAGCAGGTTTAGTTGATAGA
ACAAGAAAACCTATTGAAGACGCTTTAAGAGAAGCTAAAATTACCGCAAAAGACTTGCATGAAGTTCTATTGG
TCGGAGGTTCAACAAGAATACCTGCAGTTCAAGACATGGTTAA---
AAGAACTTTAGGAAAAGAACCAAACAGAACAATCAATCCAGATGAAGTTGTTTCGATTGGTGCTGCAATTCAA
GGATCTGTTTTAGCTGGTGATATTGATAATGATTTAACAGACCCAGTTACACTAGCTCATTTATTAAAATACGA
TACAGCATTTGGAACACT---AAAAGTT---GATGTTGAAGCT------------AAAGAAAATGC---
TATCGTAGTTAACGGAAAAGAAATTAA-----------------
AGTTTTTGCAGAAAAAGATCCAGAAGCTTTACCATGAAAAGCTCTTGACATTGACCTTGTTATTGAATGTACAG
GTTTCTTTGTTAAAAGAGAAGGAGCAGGAAAACACTTAAAAGCAGGAGCTAAAAAAGTTGTTGTTTCTGCACC
TGCAGGAAG---
TGATGTTAAAACAATCGTTTATAACGTTAACCACAAAACACTTAATGTTAATGATGACATTATTTCTGGTGCTTC
ATGTACAACAAACTGTTTAGCACCAGTAGTTAAAGTTTTAGTTGATAACTTTGGTTTAGAATCAGGATTTATG

>*Mycoplasma mobile*

AATGATTAATCGGAAAAATTAAATTAGAACAT---AAATATGATGTTTCAAAAG-------------------------------------
----

ATAAAATGGCAATGGCTAGATTAAAAGAAGAAGCAGAAAAAGCAAAAATTAATTTATCTACAACATCTACAAC
ATCAATTAATTTACCCTTTTTAGCAGTAACAGATTC---

AGGACCTATTAATGTTGAAGTCGAATTAAAAAGAAGTGATTTTGAAAAAATGACTCAACATTTAGTTGAAAGA
ACAAGAAAGCCTGTTAGAGATGCTTTAAAAGAAGCAAAATTAAAATCAGAAGATTTACATGAAGTTTTACTTG
TAGGGGGTTCAACAAGAATTCCTGCTGTTCAAGAAATGTTGCA---
ACACGAGTTGAACAAAAAACCAAATCATAGTATTAATCCAGATGAAGTAGTAGCAATTGGTGCTGCTATTCAA
GGGGCTGTTCTTTCTGGAGATATTAATAATGACTTAACAGATGCTAATACATTAGCACATTTATTTAAATATGA
TTCTATTTATGGAAATTTTAATGGGTCAATAAAAGTTGATGAA------------AAAGCTGAATC---
ATTAATTATTAATGGTCACAAAATCAG------------------
AATTTTGTCAGAAAGTGATCCTTTAAAATTACCTTGAGGTGATTTAGGAATTGATTTAGTTATTGAATCAACAG
GAAGATTTGCAACTAAAGAACAAGCAAGTCAACATTTAAAATCAGGAGCTAAAAAAGTTTTAATTTCAGCTCC
TGCTAAAGGCGCAGGAATTCCAACAGTGGTTCATAATGTAAATCACCAAATTTTAAATGCAAGTGATACAATT
ATTTCTACAGCTTCATGTACAACAAATTCATTAGCACCAGTTGCACATGCAATAAATAAAGAATTTGGAATAGA
ATCAGGTTTAATG

>*Mycoplasma synoviae*

AATGATTAACTAAAGAAATAAAAACAAAATAC---AGCTACGATGTATCTAAAG------------------------------------
----
ATAAATACGCTTTAGCTCGTTTAAAAGAAAACGCTGAAAAAGCTAAAATTGATCTATCAAATCAATCAGTTGTG
CAAATTAATATTCCATTTTTAGCAATGTCAGCTAA---
TGGGCCAATCAACGTTGAGCTTTCTCTAAAAAGAAGTGAATTTGAAGCAATGACTTCACATTTATTAGATAGA
ACCAGAAAACCTATCGAAGACGCTCTTAAAGAAGCAAAACTAAGTGCTAATGACATTCACGAAGTGCTTTTAG
TAGGTGGATCTACTAGAATGCCAGCGGTGCAAGATATGGTTAA---
AAGAACTTTAGGAAAAGAACCTAACCGTTCAATTAACCCTGACGAAGTTGTATCTATAGGAGCTGCTATCCAA
GGAGGAGTGCTAGCCGGACATATCGATAATGATTTAACTGATGCTAAAACTCTAGCTCACCTGCTAAAATACG
ATACAGCTTTCAAAAAATT---ACAATTT---TCAGTAGAAGAA------------AAAGATAGCTC---
ACTATGAGTTAATGGAAAAGAAATTAA------------------
AGTTTTTGCTGAAAAAGATCCATCAAATTTACCATGAAAAGATCTAGGAGTAGATTTAGTAGTTGAATCAACT
GGATTTTTCACTAAAAAAGACCTTGCATCTAAGCATTTAGAAGCCGGAGCTAAAAAAGTTTTAATTTCAGCGCC
AGCAGGAAG---
CGATCTTCCTACTGTGGTTTATAACGTAAACCATAAAACTCTAAAAAGTAGTGATACCGTGATTTCTGCTGCAT
CATGTACTACAAACTGCCTTGCGCCTGTGGTAAAAGTTTTAGTTGAAGAATTCGGTCTAAAATCAGGATATATG

>*Mycoplasma capricolum*

AATGATTACTAGGTAAAATTAAAGCTGAATAC---AATATTGATTTATCTAAAG--------------------------------------
----
AAAAAATGGCTTTACAAAGATTAAAAGATGAAGCTGAAAAAGCAAAAATTAATTTATCTAGCCAATTAGAAGT
TGAAATTAATTTACCATTTATTGCAATGAATGAAAG---
TGGACCAATTTCTTTTGCAACAACTCTAACAAGAAGTGAATTTAACAAAATTACAAAACATTTAGTTGACTTGA
CTATTCAACCAGTTAAAGATGCTTTAAGTGCTGCTAAAAAAACTCCAAGTGAAATTAATGAAGTTTTATTAGTT
GGTGGGTCAACAAGAATACCTGCTGTTCAAGAATTAGTAAA---
AAGTTTATTAAATAAAGAACCAAATAGATCAATTAATCCAGATGAAGTTGTTGCTATGGGTGCTGCTGTGCAA
GGTGGAGTTTTAGCTGGTGAAGTTACTAATGATTTAACTGACACTAAAACACTAGCATATTTACTTGAATTTGA
CACAGCCCAAGGAATTTTTTGCGAAGAT---GAAATTTCATAT------------ACTGATAATTC---
AATTATAGTTAAAGGAAAAGAAGTTAA------------------
AGTATTTGCTGAAAAAGATGCTGCTAACTTACCATGATCTGATTTAAAAATTGATTTAGTTGTTGAGTCAACTG

GATTTTATACAGATAAAGAAAAAGCTTCAGCTCATATTAAAGCAGGTGCAAAAAAAGTTATTATTTCAGCACC
AGCAACTGG---
AGATTTAAAAACTATTGTTTATGGGGTTAACCACAAATCATTAACAAGTGATGATGTAATTATTTCTGGAGCTT
CATGTACAACTAATTGTTTAACACCATTTACTAAAGCTTTAGATGACACATTTACTATTAAAAAAGGTTTTATG

>*Mycoplasma mycoides* Small Colony

AATGATTACTAGGTAAAATTAAAGCTGAATAC---AATATTGATTTATCTAAAG--------------------------------------
----

AAAAAATGGCTTTACAAAGATTAAAAGATGAAGCAGAAAAAGCAAAAATTAATTTATCTAGTCAATTAGAAGT
TGAAATTAATTTACCATTTATTGCAATGAATGAAAG---
TGGACCAATTTCTTTTGCAACAACTCTAACAAGAAGTGAATTTAACAAAATTACAAAACATTTAGTTGATTTAA
CTATTCAACCAGTTAAAGATGCTTTAAGTGCTGCTAAAAAAACTCCAAGTGAAATTAATGAAGTTTTATTAGTA
GGTGGATCAACAAGAATACCAGCTGTTCAAGAATTAGTAAA---
AAGTTTATTAAATAAAGAACCAAATAGATCAATTAATCCAGATGAAGTTGTTGCTATGGGTGCTGCTGTTCAA
GGTGGAGTTTTAGCTGGTGAAGTTACTAATGATCTAACTGACACTAAAACATTAGCTTATTTATTAGAATTTGA
TACAGCTCAAGGAATCTTTTGTGAAGGT---GAAATCTCACAT------------ACAGATAATTC---
AATTATAATTAAAGGAAAAGAAGTAAA------------------
AGTATTTGCTGAAAAAGATGCTTCTAATTTACCTTGAAGTGAATTAAAAGTTGATTTAGTAATTGAATTAACTG
GATTTTATACAGATAAAGAAAAAGCTTCAGCTCACATTAAAGCAGGAGCTAAAAAAGTGGTTATTTCAGCTCC
AGCAACTGG---
AGATTTAAAAACTGTTGTTTATGGAGTTAACCACAAATCATTAACTAGTGATGATGTAATTATTTCTGGAGCAT
CATGTACAACTAACTGTTTAACACCATTTACTAAGGCTTTAGATGATGCATTTACTATTAAAAAAGGATTTATG

>*Mesoplasma florum*

ACTGATTAATTGAAAAAATTAAAACTGAAAGT---GGTGTTGATTTAAAAAACG--------------------------------------
----

ATAAAATGGCATTACAAAGATTAAAAGATGAAGCTGAAAAAGCAAAAATTAATTTATCAAGTCAATTAGAAGT
TGAAATTAACTTACCATTTATTGCAATGAATGAAAA---
TGGACCAGTTTCATTCTCAACTCAATTTTCAAGAACAGAATTTGACAAAATTACAAAAGATTTAGTTGAAAGAA
CTTCTAAACCAGTAAAAGATGCATTACAAGCAGCTAAATTAAGTGCTAGTGATATTGATGAAGTTTTACTAGTT
GGTGGATCAACAAGAATACCAGCTGTTCAAAAAATTGTTAA---
AGAATTATTAGGAAAAGAACCAAACCGTTCAATTAACCCAGATGAAGTTGTAGCTATGGGTGCAGCTATCCAA
GGTGGGGTTTTAGCGGGAGATGTTACTAACGATTTAACAGACACAAAAACATTAGCTTACTTATTAGAATTTG
ACTCAGCACAAGGAAAATTCCAAGAAGGA---AAAATTTCATAC------------ACAGATAACTC---
AATTATCGTTAATGGAAAAGAAATTAA------------------
AATCTTTGCTGAAAGAAATGCAGCTGATTTACCTTGAGGTAAATTAGGAATCGATTTAGTAATCGAATCAACA
GGATTCTACACAGATAAAGAAAAAGCATCAGCACACTTAACAGCTGGAGCAAAAAAAGTTATTATTTCAGCAC
CTGCTACAGG---
AGAAATGAAAACAATCGTTTATGGTGTAAACCACAAAAACTTATCAGCAGAAGACGTAATTATTTCAGGAGCT
TCATGTACAACAAACTGTTTATCACCTGTTGCTAAAATCATGGATGAAAAATTTGGAATCGTTAAAGGAAAAAT
G

>*Mycoplasma penetrans*

ACTGAATTGTTGAAGAAGTTAAGAAGAATGAT---AAAGTTGATTTATCAAATG------------------------------------
----

ACAAAATGGCAATGCAAAGATTAAAAGATGCTGCAGAAAAAGCTAAGATTGATCTTTCGGGATTAAAAGAAG
TTGAAATTAGTTTACCTTTTATTGCTATGACTGAAAG---
TGGCCCATTAAATGTAGATCTAAAATTAACAAGAGCTAAATTTGAAGATCTAACAAGAGACTTATTAGAAAGA
ACTATCAAACCAGTTGAAGATGCTTTAAAAGAAGCTAAATTATCAGCAAGTGATATTCATAAAGTTTTATTAGT
TGGTGGTTCTACTAGAATGCCAGCAGTTGAAGAACTAGTTAA---
ATCTAAGCTAGGAAAAAGTCCTGATAAAAACATCAATCCTGATGAAGTAGTTGCTGCAGGTGCTGCTATTCAA
GGTGGTGTATTAATGGGGGATGTTAATAATGACTTGACTGATGCTAAAACATTATGTCACTTATTAAAATATG
ACACGGCTCATAGAACATTTAAAGGGAAG---TTATCATATGAT------------GAAAATAACAA---
TTTAATCATTGATGGAAAGAAAATCCC-----------------
AATCTTAGCTGAAAAAGATCCAGCTAACTTACCTTGAGCAAAATTAGGTGTTGATATTGTTGTAGAATCTACTG
GTAGATTTGTTGATGAAGAAGGTGCATCTAAGCACTTAAAAGCTGGAGCTAAAAAAGTAATTATCTCTGCTCC
TGCTAAAGG---
AAACATTCCTACAGTTGTTTACAACGTAAACCACCAAACACTTAAAGCTACTGACAAAATTGTTTCAGCAGCAT
CATGTACTACAAATGCATTAGCACCAGTTGCAAATGTATTAAGTAAAGAATTTGGTATTAAATGAGGATTCAT
G

>*Mycoplasma cavipharyngis*

ATTGATTAATTACTGAAATCCGTAATGAACAC---AGTATTGATTTAACTAAAG--

ATAAAATGGCAATGCAACGATTAAAAGATGCTGCAGAAAAAGCCAAAATTGATTTATCAGGTTTAACATCAGT
TCAAATTCTTTTACCATTTTTATCAATGAGTCATGG---
TCAACCATTAAATGTTGATAAAACTTTAACTAGAACTCATTTTGAAAATTTAACTAAAGATTTATTGAACCGAAC
TATTAAACCATTAGAAGACGCTTTAAAAGAAGCAAAAATTGAAACTAGTGAGTTAAACCAAGTTTTATTAGTT
GGTGGTTCAACAAGAATGCCTGCTGTTCAAGCAATGGTTGA---
AAAAATTACTGGTCGCAAACCAAATTTATCAATTAATCCTGATGAAGTAGTAGCATTAGGTGCTGCTGTACAA
GCTGGAGTTTTAAGTGGATCAGTTAATAATGACTTAACTGATGCAAAAACTCTTGCTCATTTACTAAAATATGA
TAGTGCTCAAGGTATTTTAAAAGGTTTT---TGTGTTGAAACT------------GAAGGTGATAA---
TTTAAAAATAATTAATCATCAAACTAATGCAGTAACTTTAGTGCCTGTTTACTCTGAACGTGATCCTAAAAATTT
ACCATGAGTTAAATTAGGAGTTGAAGTTGTTTTAGAATGTACTGGTTTATTTACTAAAAAAGAAGCTGCTGAA
CAGCATTTGATTGCTGGTGCTAAAAAAGTATTAATTTCAGCACCTGGTACAGG---
TGATATGAAAACAGTTGTTTATAATGTTAATCACGACATTTTAACTCCATCTGATCAAATTGTTAGTGCTGCTTC
ATGTACAACTAATGGTTTAGCTCCTGTAGTTTATTTTTTAGATCAAGCTTTTGGAATTAAATCAGGAACAATG

>*Mycoplasma gallisepticum*

AATGGATCATTGCTGAAATCAAAAAAGATCACCCATCATTAGACCTTAAGTCTG-----------------------------------
----

ATAAGATGGCAATGCAAAGATTAAAAGAAGCTGCTGAAAGAGCTAAGATCGAACTATCAGCTCAATTAGAAA
CACTAATCTCATTACCATTCATCGCAGTTACTCCTGA---
AGGTCCAGTAAACGCTGAATTAACTTTATCAAGAGCTAAATTCGAAGAATTAACTAAAGACTTACTAGAAAGA
ACAAGAAACCCAATTGCTGACGTATTAAAAGAAGCTAAGGTTGATCCTAGTCAAGTTGATGAAATTCTTTTAG
TAGGTGGTTCTACAAGAATGCCTGCAGTACAAAAATTAGTTGAATCAATGATTCCTAATAAAGCACCAAACCG
TACGATTAACCCTGACGAAGTAGTAGCGATCGGTGCTGCTGTACAAGGTGGGGTATTACGTGGGGATGTTAA

TAACGACTTAACTGATGCTAAGACACTTGCTTATTTACTAAAATATGATACAGCTCACGGTAAGTT---
GAGTCAC---AACGTGAGTTCA------------ACTGATAGTGA---GATCGTAGTTGGTAAACAAAAAATTAA-----------
----

AGTTTACAGTGAAAAAGATCCTACACAAATTCCATGAAAGAAACACAAAGTTGATTTAGTAGTTGAATCAACT
GGTCGTTTTTTAACTCAAGAAGCTGCTAGTGCACACCTTAAAGGTGGTGCTAAGAAAGTAGTTTTATCAGCTCC
AGCTAAAGAAAAGAACGTGAAAACTGTAGTTTACAACGTGAACCACACTGAAATTAAACCAGATGATACTGTA
ATTTCAGCTGCTAGTTGTACAACTAACTGTTTAGCTCCACTTGTTAAAGTGTTAGAAGACAAGTTCGGAATTAA
AGTTGGTTATATG

>*Mycoplasma fastidiosum*

ATTGAATAGTTGAAAACATTACTAAAGATCATCCGAATTTAAAAATTCGTGAAG-----------------------------------
----

ACAAAATGGCTATGCAACGTTTAAAAGAGGCTGCTGAAAGAGCTAAAATTGAGTTATCTGCTCAATTAGAAGT
TAATGTTTCATTACCATTCATTGCTGTAACTGAATC---
TGGTCCAGTTAACTTTGATATGCAATTATCTAGATCTAAATTTGAACAATTAACTAAGGATTTAGTTGAAAGAA
CAAGAAACCCAATTAAAGACGTATTAACAGAAGCTAAAGTTGATCCTTCTCAAGTTGATGAAATTTTATTAGTT
GGTGGTTCTACAAGAATTCCAGCTGTTCAAGCTTTAGTTGAATCAATGGTTCCTAATAAGAAACCTAACCGTAC
TATTAACCCCGATGAAGTTGTTGCAGTAGGTGCTGCAGTTCAAGGTGGTGTATTACGTGGTGATGTTAATAAT
GATTTAACTGACGCAAAAACTTTAGCTCATTTATTGAAGTATGATACAGCTCATGGTGCATTGCCAAATTTT---
GTTACTGTAAAT------------GATAAAAATAA---TATTTCAATAGATGGTAAAGAATTAAA------------------
GGTTTTTGCTGAAAAAGATCCATCTAAATTACCTTGAAAAGATTTAGGAATTGATTTAGTAGTTGAATCAACTG
GATTATTTTTAACAAAAGAAAAAGCACAATTACATATTGATGCAGGCGCAAGAAAAGTTTTATTGTCAGCTCC
AGCCAAAGAAAAAAATATTAAAACTGTTGTGTTTAATGTGAATCATGAAATTATTAATTCAGATGATCAAATAA
TTTCTGCTGCTTCTTGTACAACTAACTCATTAGCACCTGTTGTTAAAGTTTTAGAAGATAATTTTAAGATTGTTG
CAGGAACAATG

>*Mycoplasma genitalium*

AATATATCTCAGCCTACATTGCCAAAGAACACCAGGGTTTAAACTTATCAAAAG-----------------------------------
----

ATAAGATGGCAATGCAACGGCTTAAAGAAGCAGCTGAACGTGCTAAGATTGAACTTTCCGCTCAACTTGAAAC
GATTATTTCTCTACCATTTTTAACTGTTACCCAAAA---
AGGTCCTGTTAACGTTGAGTTAAAACTAACCCGTGCTAAGTTTGAGGAGTTAACAAAACCACTACTTGAAAGA
ACAAGAAACCCTATTTCAGATGTTATCAAGGAAGCTAAGATTAAACCTGAAGAGATTAATGAAATTCTTTTAGT
TGGTGGTTCTACAAGGATGCCTGCAGTTCAAAAGCTAGTTGAATCAATGGTACCAGGTAAAAAACCAAACCGT
TCTATTAATCCTGATGAAGTTGTTGCTATTGGCGCTGCTATTCAAGGTGGGGTTTTACGTGGTGATGTTAATAA
TGATTTGACCCAACCTGAAGTTTTAGCGCACCTGTTGAAATATGATTCAGCTCATGGTGAATT---GAAAAGA---
AAGATTACTGTT------------AAACAAAACAT---CTTGCAAATTGATAGAAAAAAGGTTTA------------------
TGTTTTTAGTGAAAAAGATCCCCAAAATTTACCTTGGGATGAACATGATATTGATGTAGTAATTGAATCAACTG
GTAGGTTTGTAAGTGAAGAGGGTGCTTCTCTCCATTTAAAAGCAGGTGCTAAAAGAGTAATTATTTCCGCACC
CGCTAAAGAAAAAACTATCAGGACAGTTGTTTACAATGTTAATCACAAAACCATTAGTAGCGATGATAAGATC
ATCTCAGCAGCTAGCTGTACTACTAACTGTTTAGCACCATTAGTTCATGTACTTGAAAAGAACTTTGGGATTGT
TTATGGAACGATG

>*Mycoplasma pneumoniae*

AGTTCATCTTAGCGCACATTGCCCAAGAACACAATGGGCTTAACTTGTCCAATG-----------------------------------
----

ACAAGATGGCTATGCAACGCTTAAAGGAAGCGGCTGAACGTGCTAAGATTGAACTTTCCGCCCAACTAGAAG
CAATTATCTCTTTACCGTTCTTAACGGTTACCGAAAA---
GGGTCCGGTAAACGTTGAACTTAAGCTAACCCGTGCTAAGTTTGAAGAAATTACCAAACAATTACTAGAACGT
ACTCGCAACCCAATTTCGGATGTTTTACGTGAAGCCAAGATTAAACCAGAAGAAATTAATGAAATCTTGTTGG
TGGGTGGATCGACCCGGATGCCAGCAGTGCAAAAACTAGTGGAATCAATGGTACCAGGACACAGTCCAAACC
GCTCAATTAACCCGGATGAGGTGGTAGCCATTGGTGCTGCCATCCAAGGGGGTGTGTTACGCGGTGATGTAA
ATAACGACCTCACCCATCCCGATACTTTAGCACACTTACTAAAGTACGACTCCGCTCACGGTGAGTT---
TAAGAAA---AAGGTAGTAGCT------------AAGGACAACAC---CTTAATGATTGACAAAAAGAAGGTGTT----------
----

AGTGTTTAGTGAAAAAGATCCTGCTAACCTGCCATGAGCAGAACACAATATTGATATTGTGGTTGAATCCACT
GGTCGCTTTGTTAGTGAAGAAGGTGCTAGTTTGCACTTGCAAGCTGGTGCTAAACGCGTGATTATTTCCGCTC
CAGCTAAACAAAAAACAATTAAAACCGTGGTTTACAACGTGAACCACAAGATTATTAACGCTGAGGACAAAAT
TATCTCAGCTGCTAGCTGTACCACCAATTGCTTAGCACCAATGGTACACGTCTTGGAAAAGAACTTCGGCATCT
TACACGGTACCATG

>*Acholeoplasma laidlawii*

AATTCTTAGCAGCTGAATTTAATAAAGAACAC---GGTATTGATTTACTAAAAG-------------------------------------
----

ATAAAATGGCTTTACAACGTCTACGTGATGCAGCAGAAAAAGCTAAGAAAGAATTATCAGGTGTAACAACAA
GTTCTATTTCTCTACCATTTATTACAATGGGAGCAGC---
AGGTCCGTTACACTTTGAATATAACTTAACGCGTGCTAAATTTAATGAATTGACTAAAGATTTAGTAGATCGTA
CAATGGTATCTGTTAGACGTGCTTTAGCTGATGCTAAATTAACAAACTCTGATATTAATCAAATCTTATTAGTTG
GTGGTTCAACAAGAATTCCAGCTGTTCAAGAACTTGTAAC---
AAAAGAATTTGGTAAAGAACCTAATAGATCGGTAAACCCTGATGAAGTTGTAGCAATGGGTGCTGCAATCCA
AGGTGCTGTTTTAGCAGGGGATGTTAACAACGATTTAACTGATACAGAAACTCTTGCTTACCTTTTAAAGTATG
ATACAGCTCAAGGTCCTTTCAAAGGTCAT---GAAGTATCAACA------------AACGGAGATAA---
ATTAGTAGTTGATGGTAAATCAATCAC------------------
TGTTTACGCTCAAAAAGATCCATCTGAGTTACCATGGGGTACACTTGGTGTTGACGTAGTATTAGAGTCAACA
GGATTATTCACATCAGCTGAAAAAGCTGGTTTACACATCAAAGCGGGTGCTAAGAAAGTTGTAGTTTCTGCAC
CAGCAACAGGTGAAGGCGTTAAAACTGTTGTTTACAACGTAAACGATGACATCTTAGATGGAACTGAAACAAT
CGTTTCAGCTGCATCATGTACAACTAATGCTTTAGCTCCTTTAGCAAAAGTTTTAGATGACAACTTTGGTATCGT
TAAAGGTTTCATG

>*Candidatus* Phytoplamsa austaliense

ATTTTTTAGTTAAAGAATTCAAGAAAGATAAC---GGAGTTGATTTATCAAAAG-------------------------------------
----

ATAAAATGGCAATGCAACGTTTAAAAGATGTTGCTGAAAAAGCTAAAAAAGAATTAAGTGGTGTCACTTCTAG
CCAAATTTCTTTACCTTTCTTAACTATGAGCGCGTC---
AGGTCCTTTGCATTTAGAATACAATATGACACGTGCGAAGTTTAATGAATTAACTAAAGATTTAATTGATCGTT
GTTTAGGTCCTGTTAAACGTGCTTTGAGTGATGCTAAATTAACTATTGAAAAAATCGATCAAGTTCTTTTAGTG
GGTGGTTCTACTCGTATTCCTGCTGTTCAAGAATTAGTTAA---
AAAAGAATTACAAAAAACTCCTAATAAAAGTATTAATCCAGATGAAGTAGTTGCAATTGGTGCTGCTATTCAA

GGAGGTATTTTAGCAGGAGATGTGAATAATGATTTATCATCTTTAGAAACTATTTCTTATTTGCTTAAATATGAT
AGTATACAAAGACCTTATGAAGTTGAT---GCTGTTAGTTTT------------GAAGGAAAAAA---
TTTAATAGTTAAAGGTGAACAAATTCC-----------------
TGTTTTTCAAGAAAAAAACCCCCAAGATTTGCCTTGGAAAGAATTAGGGGTTGATATTGTTTTGGAATGTACA
GGTTTTTTCACCGATAAAGAAAAGGCATCCTTACATTTAAAAGCAGGCGCTCGCAAAGTTTTAATTAGTGCTCC
TGCGACAGG---
AGATGTTAAAACTATTGTTTATAACGTTAACGATCATACTTTAAATGAAAATGATATTATTGTTAGTGGAGCAT
CATGTACTACTAACTGTTTAGCGCCAATTGTCAAAATTTTAAATGATAATTTCGGTATTAAACAAGCTTTTATG

>Aster Yellows Witches’ Broom Phytoplasma

ATTTCTTAGTGCAAGAATTCAAAAAAGAAAAT---AGTGTTGATCTTTCCAAAG-------------------------------------
----
ATAAAATGGCAATGCAAAGATTAAAAGATGCTGCTGAAAAAGCTAAAAAAGAATTAAGTGGTGTTGCTTCTTC
ACAAATTTCTTTGCCTTTCTTAACAATGAGCGAAGC---
AGGTCCCCTTCATTTAGAATACAATATGACTCGTGCTAAATTCAATGAACTTACTAAAGATTTAATTGATCGTTG
TTTGGCTCCTGTAAAACGTGCTTTAAGTGATGCCAAATTAGATATTAAAAAAATCGATCAAGTGCTTTTAGTGG
GTGGTTCTACTCGTATTCCTGCAGTTCAGGATTTAGTTAA---
AAATGAATTGAATAAAACTCCTAATAAGAGCATTAATCCTGATGAAGTAGTTGCTATTGGAGCTGCTATTCAA
GGAGGAATTCTATCAGGAGAAGTTAATAATGATTTAGCAACTTTAGAAACTATTTCTTATTTATTAAAATACGA
CAGCATTCAAAAACCTTACAAATTAGAC---GCTGTTAGTTTT------------GAAGACAACTA---
TTTAGTAGTTGAAGGACAAAAAATTCC-----------------
TGTATTTCGAGAAAAAAACCCTCAATGTTTACCTTGGAAAAAACTAGGAGTAGATATTGTTTTAGAATGTACA
GGTTTTTTTACTAGCAAAGAAAAAGCATCTTTTCATTTAGAAGCTGGAGCTAAAAAAGTGTTAATTAGTGCTCC
TGCTATTGG---
GGATGTAAAAACAGTTGTTTACAATGTTAATGATCAAATCCTAACCAAAGAAGACGCCATTGTAAGTGGGGCT
TCGTGTACTACTAACTGTTTGGCGCCTGTTGTTAAAGTCTTAAATGATAATTTTGGCATCAACCAAGCTTTTATG

>Onions Yellows Phytoplasma

ATTTCTTAGTGCAAGAATTCAAAAAAGAAAAT---AGTGTTGATCTTTCCAAAG-------------------------------------
----
ATAAAATGGCAATGCAAAGATTAAAAGATGCTGCTGAAAAAGCTAAAAAAGAATTAAGTGGTGTTACTTCTTC
ACAAATTTCTTTGCCTTTCTTAACAATGAGCGAAGC---
AGGTCCACTTCATTTAGAATACAATATGACTCGCGCTAAATTCAATGAACTTACTAAAGATTTAATTGATCGTT
GTTTGGCTCCTGTAAAACGTGCTTTAGGTGATGCTAAATTAGATATTGAAAAAATTGATCAAGTGCTTTTAGTG
GGTGGTTCTACTCGTATTCCTGCAGTACAAGATTTAGTTAA---
AAATGAATTGAAAAAAACTCCTAATAAGAGTATTAATCCTGATGAAGTAGTTGGTATTGGAGCTGCTATTCAA
GGAGGAATTCTATCAGGAGACGTTAACAATGATTTAGCAACTTTAGAAACTATTTCTTATTTATTAAAATACGA
CAGCATTCAAAAACCTTACAAATTAGAC---GCTGTTAGTTTT------------GAAGACAACTA---
TTTAGTAGTTGAAGGACAAAAAATTCC-----------------
TGTGTTTCAAGAAAAAAATCCTCAAGATTTACCTTGGAAAAAACTAGGAGTAGATATTGTTTTAGAATGTACA
GGTTTTTTTACTAGCAAAGAAAAAGCAGCTCTTCATTTAGAAGCTGGAGCTAAAAAAGTGTTAATTAGTGCTCC
TGCTACTGG---
GGATGTAAAAACAGTTGTTTACAATGTTAATGACCAAATACTTACTAAAGAAGATGCCATTGTAAGCGGGGCT
TCTTGCACTACTAACTGTTTGGCGCCCGTTGTTAAAGTCTTAAATGACAACTTCGGCATCAACCAAGCTTTTATG

>*Clostridium perfringens*

ATTATATAGCAGAAGACTTTAAAGCTCAAAAC---GGAATTGATTTAAGACAAG-------------------------------------
----
ATAAAATGGCTCTTCAAAGATTAAAAGAAGCTGCTGAAAAAGCTAAAATTGAGTTATCATCATCAACTCAAAC
ATTAATCAACTTACCATTTATAACTGCTGATGCAAC---
TGGTCCAAAACACATAGATATGACATTAACAAGAGCTAAATTCAATGAATTAACTCATGACTTAGTTGAAAGA
ACAATCAACATAATGAAAGAAGCCTTAAAATCAGGTAATGTTTCATTAAATGATATAGATAAAGTAATCTTAGT
TGGTGGATCAACAAGAATACCAGCAGTTCAAGAAGCTGTTAA---
AAACTTCACTGGAAAAGAACCTTCAAAAGGAGTTAACCCAGATGAGTGCGTAGCAATGGGTGCTGCTATCCA
AGCTGGTGTATTAACTGGTGATGTTAACAACGACTTAACTGATGCTAAGACTTTAGCACACTTATTCAAATACG
ATTCAGCACAAGGAAGATT---CAATGGT---GAAATAGAAGTT------------AAAGAAGGAGC---
TTTCGTAGTTAACGGAAAAGAAATCAA------------------
AGTAACTGCTAAAAGCAACCCTGCTGAATTACCATGGGGAGAATTAGGAGTAGACGTAGTATTAGAGTGTAC
TGGATTCTTCGCATCAAAAGAGAAAGCTTCAGCTCACTTAACTGCTGGTGCTAAAAAAGTTGTTATCTCAGCTC
CTGCTGGAAA---
CGACCTACCAACAGTTGTTTACAACGTAAACCACGATATATTAGATGGAAGCGAAGATGTTATCTCAGGTGCT
TCATGTACTACAAACTGCTTAGCTCCAATGGCTAAAGCTTTAAATGATAACTTCGGATTAAACAAAGGTTTCAT
G
